# A high precision finite-element forward solver for surface nuclear magnetic resonance incorporating conductivity changes and surface-topography variations

**DOI:** 10.1371/journal.pone.0264235

**Published:** 2022-03-17

**Authors:** Hanbo Chen, Bin Xiong, Chi Zhang, Ziyu Cheng

**Affiliations:** 1 College of Earth Sciences, Guilin University of Technology, Guilin, China; 2 Department of Geology, University of Kansas, Lawrence, Kansas, United States of America; Universiti Brunei Darussalam, BRUNEI DARUSSALAM

## Abstract

Surface nuclear magnetic resonance (SNMR) is a geophysical method that can be used directly for detecting groundwater resources, and it has attracted the attention of many scholars. In this paper, we propose a new effective algorithm for numerical modeling of 3D SNMR data for arbitrary topography in a conductive medium. We adopt a total-field algorithm for solving the quasi-static variant of Maxwell’s equation and handle a complex-shaped loop source by discretizing the transmitter into electric dipoles, which can be further easily discretized into electric dipoles along the three directions of the Cartesian coordinate system. To solve the 3D SNMR forward-modeling problem quickly and accurately, a new element-integration system based on a new symmetric orthogonal rule is used for calculating the sensitivity (i.e., kernel) functions of all elements. The new rule is based on a special arrangement involving a cubic close-packed lattice structure and is characterized by fast convergence, positive weight, and symmetry. We apply the developed numerical algorithm to SNMR tomography of several typical hydrogeological models. The synthetic results show that higher precision can be achieved with few grids and nodes without increasing the computation time by using the new integration algorithm. In addition, we find that the topography and conductivity can affect the SNMR response, which needs to be considered while interpreting SNMR data.

## Introduction

**Fig 1:**
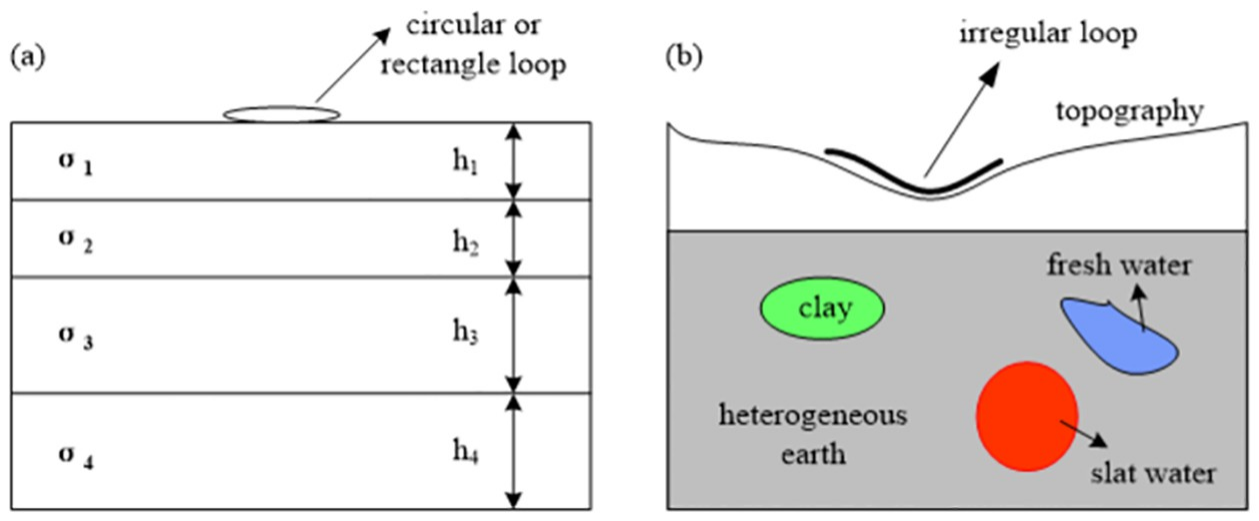
(A) The traditional SNMR numerical simulation assumes that the shape of the excitation source is a circular or rectangular ring, the topography is flat, and the underground medium structure is a horizontally layered structure. (B) Our numerical simulation assumes that the excitation source has an arbitrary shape, the topography is arbitrary, and the underground medium structure is an uneven 3D structure.

In recent years, surface nuclear magnetic resonance (SNMR) has been promoted for groundwater exploration on land; its advantages include nondestructive detection, high efficiency, and unique solutions [1–6]. Currently, a 1D model is often used for groundwater exploration [5, 7–12]. In the conventional SNMR forward numerical simulation, to simplify the forward problem, the excitation magnetic field of the transmission coil is usually calculated with a half-space with homogeneous conductivity or an N-layer horizontally layered medium model as the background medium. Although the problem is simplified, these methods are limited to the regular loop shape and 1D conductive structure on the plane terrain (Fig 1A). Therefore, it is necessary to conduct full 3D modeling of SNMR data that considers the undulating terrain and the uneven conductivity distribution, so that we can accurately interpret the field SNMR data.

The surface topography has a strong influence on the electromagnetic field response (Sasaki, 2011) and further affects the SNMR response. A challenge in SNMR data interpretation is that the influence of topography will likely lead to serious data distortion. To accurately interpret the subsurface water-bearing structure, the effect of topography should be considered in SNMR numerical simulations. It is well known that the finite-element method (FEM) based on a tetrahedron is very suitable for solving this type of numerical simulation problems [13, 14]. In the subsurface environment, there are complex exploration objects, such as fissure and karst water, which are characterized by small-scale and uneven water distribution; therefore, it is necessary to consider the surface topography in SNMR forward modeling, which will improve the detection accuracy of SNMR.

The accuracy and efficiency of the forward calculation of the SNMR sensitivity function are essential for realistic simulations and reliable inverse models from field data. Usually, the sensitivity function of each mesh element is calculated using only the sensitivity function of the four nodes of element-by-element integration [15–17]. Because of the sinusoidal component in the kernel function, the kernel function changes sharply near the loop and requires a much denser mesh for a highly accurate forward-modeling result. This causes additional problems, such as significant increases in the required storage space and computation time. Therefore, it is necessary to conduct an in-depth study on how to calculate the kernel function quickly without reducing the accuracy.

In this study, to address the above limitations while considering the undulating terrain, the shapes of excitation and receiving coils, and the uneven conductivity distribution of the underground medium at the same time (Fig 1B), we present a new algorithm for solving the SNMR forward-modeling problem based on an electric-dipole discretization method with a finite-element technique. The new algorithm can simulate the electromagnetic field response under a 3D geological model with arbitrary coil shape, undulating terrain, and underground conductivity distribution. The forward calculation of the sensitivity kernel function of the ground magnetic resonance is directly related to the accuracy and reliability of the inversion results. We use a new symmetric quadrature rule for the tetrahedral to calculate the kernel function in the region near the coil. Compared to the conventional method of refining the mesh in the region near the transmission and reception coils, our approach improves the accuracy of the kernel function without adding too many mesh elements, and reduces the required computational memory while improving the computational efficiency.

First, we designed a horizontally layered medium model to test the correctness of the code. Subsequently, we designed an elliptical aquifer model with different depths to further verify the effectiveness of the proposed algorithm. Finally, we designed a comprehensive hydrogeological undulating-terrain model and analyzed the results from the application of the new algorithm thereby providing a comprehensive example of hydro-geophysical correlation, and highlighted the influence of the undulating terrain and conductive characteristics on the SNMR response.

## Theoretical background

### Surface nuclear magnetic resonance (SNMR)

**Fig 2:**
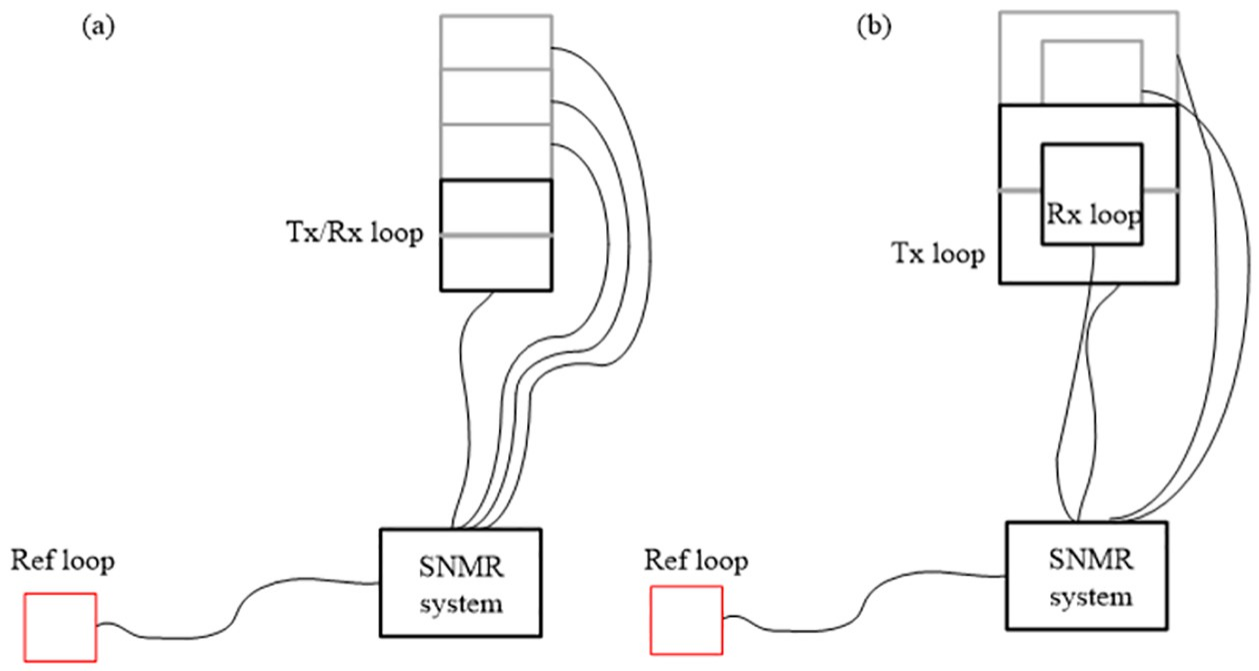
SNMR field setup. (A) Coincident loop configuration. (B) Separated loop layouts. The shapes and sizes of the transmission and reception loops can be different and one can arrange them in different ways. In general, the size and shape of the reference loop are set to be the same as those of the transmission/reception loop.

SNMR is an effective geophysical technology and has been successfully applied for directly evaluating the properties of aquifers. The setup of the SNMR method is shown in Fig 2; a complete SNMR consists of a circular or rectangular wire loop placed at the surface (Fig 2B). This loop can be used only from transmission, but it can also be used as both transmission and reception coil (Fig 2A). Assuming the magnetic field of the Earth to be static, the essence of SNMR surveys is to sample large volumes of protons from underground water. The SNMR responses are excited by protons in the underground area near the excitation coil; therefore, accurately calculating the magnetic field near the coil is crucial for improving the accuracy of the 3D SNMR forward calculation. This is discussed in the next section.

### Electromagnetic formulation

In SNMR surveys (Fig 1), the precise quantification of groundwater requires the use of a magnetic field generated by an oscillating current with frequency ranging from 1.3 to 3.7 kHz supplied to the surface excitation coil. This is similar to other electromagnetic methods in geophysics (e.g., Controlled-Source Electromagnetic Method (CSEM), Controlled-Source audio Magnetotelluric method (CSAMT)). The difference between SNMR and electromagnetic (EM) induction techniques is that the SNMR tomography needs highly accurate calculation of the magnetic field at all points in the underground space, while the latter only needs calculation of the EM fields at the receiver positions. Similarly to the EM induction techniques, we ignore the displacement current and apply the quasi-static approximation on Maxwell’s equations. We set the magnetic permeability value to be the same as that of air in free space. The frequency-domain electromagnetic problem satisfies Maxwell’s equations as follows [18]:

$$\nabla \times E=i{\omega \mu }_{0}H,$$ (1)

$$\nabla \times H=J_{s}+\bar{\sigma }E,$$ (2)

where ω is the angular frequency. *J*_*s*_ is the current density distribution.

Calculating the curl on the left-hand side and the right-hand side of Eq (1), and substituting Eq (2) into Eq (1), we eliminate the magnetic-field variable. Subsequently, we obtain the Helmholtz equation:

$$\nabla \times \nabla \times E-i{\omega \mu }_{0}\bar{\sigma }E=i{\omega \mu }_{0}J_{s}.$$ (3)

**Fig 3:**
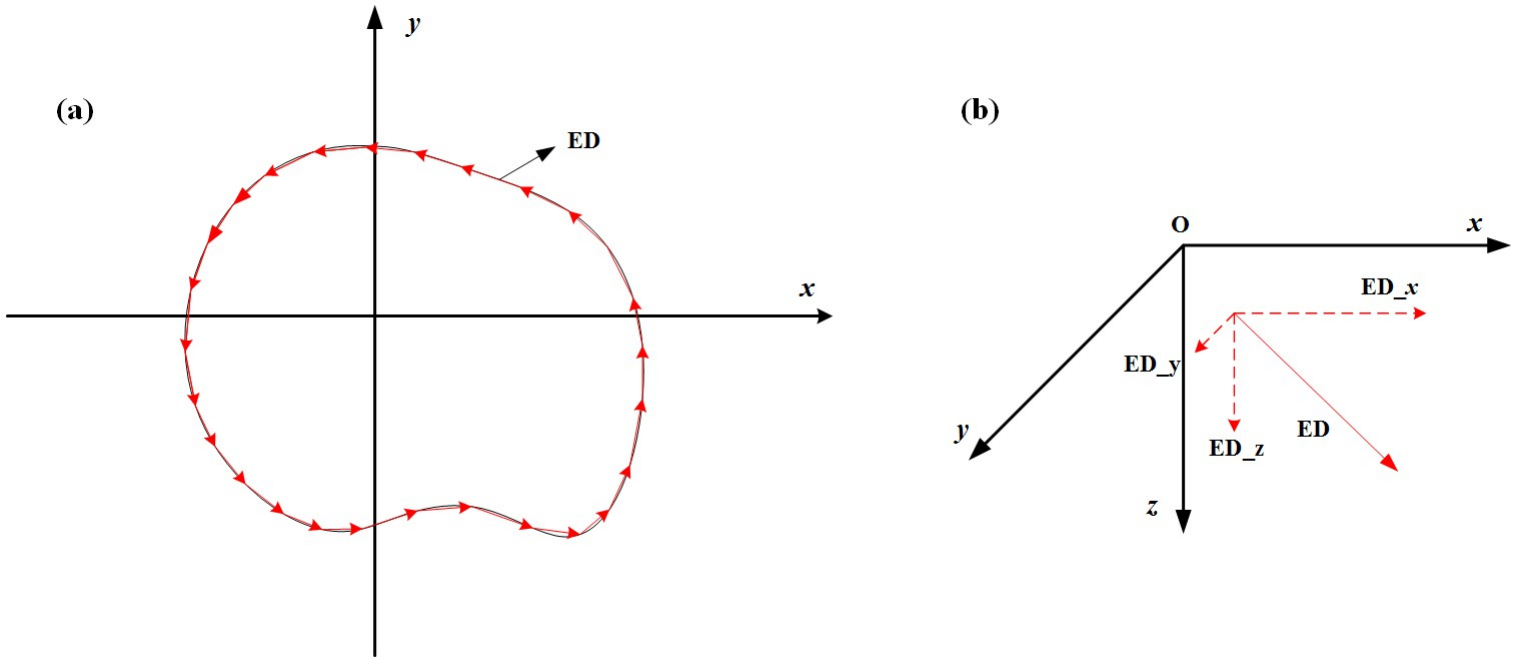
(A) A loop is decomposed into many electric dipoles. (B) An electric dipole can be further decomposed into three electric dipoles along the direction of the cartesian coordinates.

**Fig 4:**
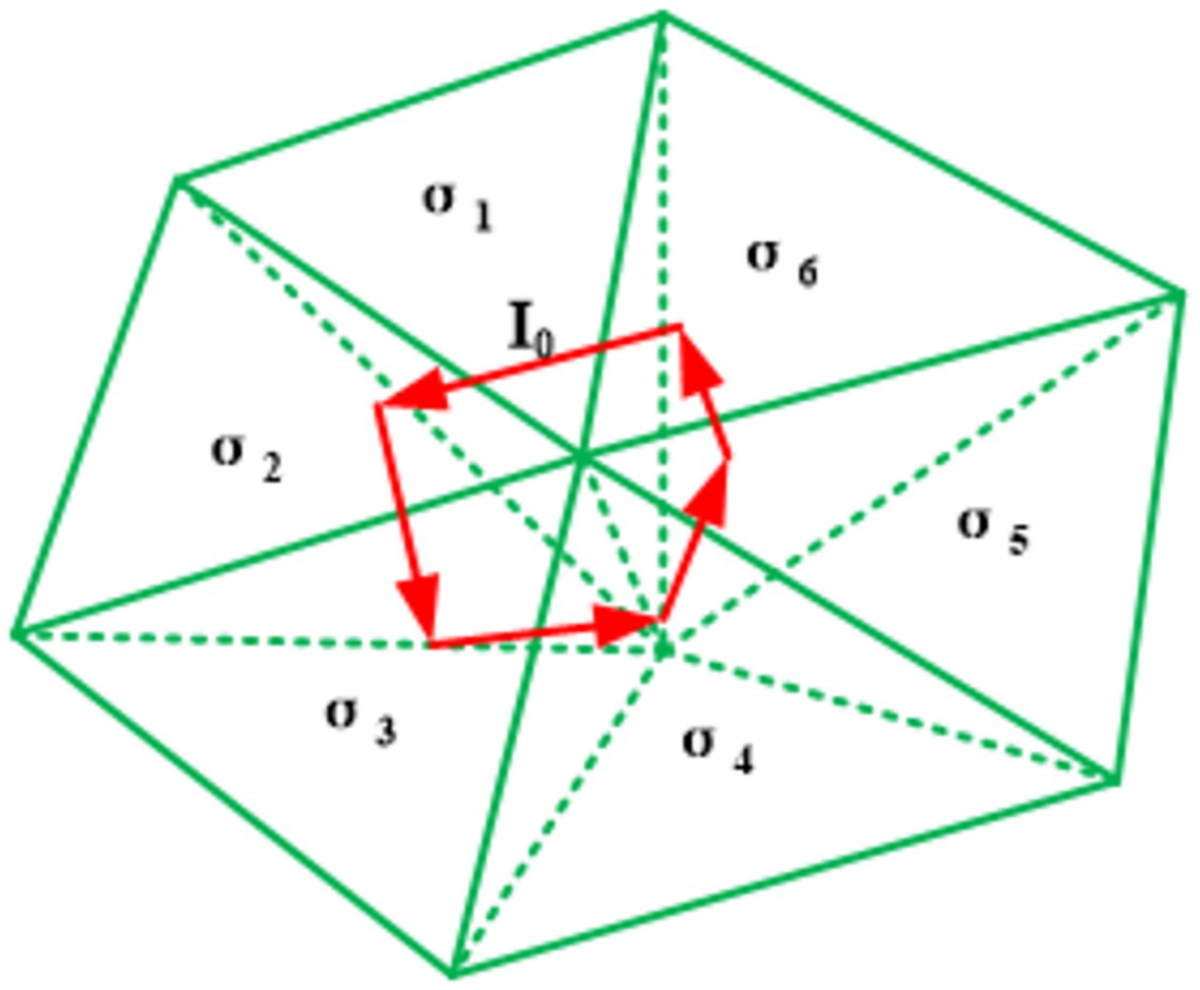
Position relation of sources in the mesh.

**Fig 5:**
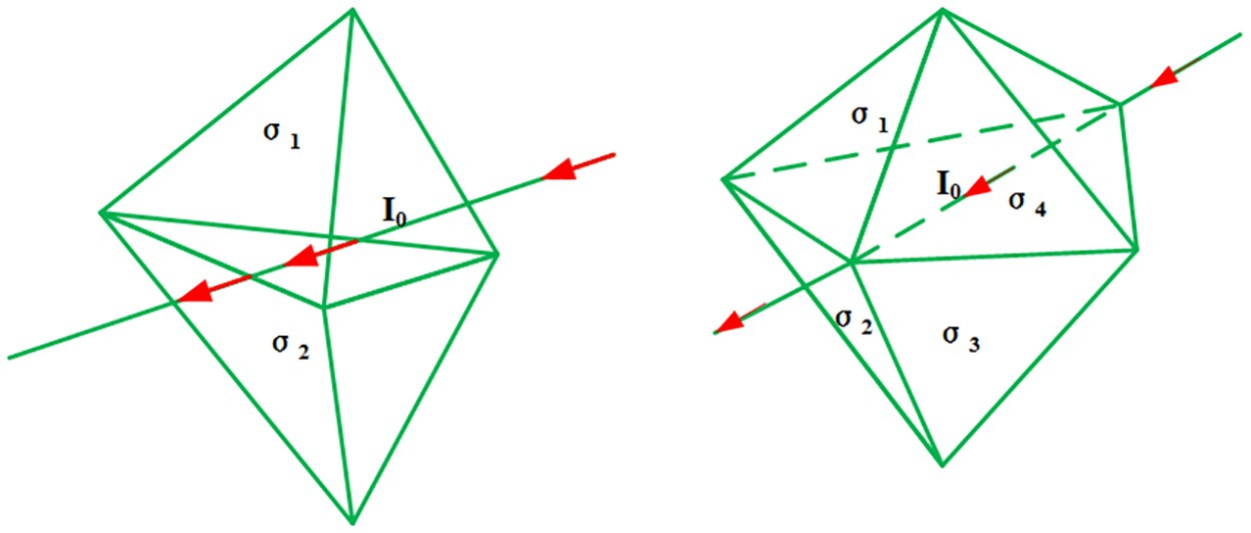
Contact relationships of the source with the (A) surface and (B) edge of the tetrahedral element.

**Fig 6:**
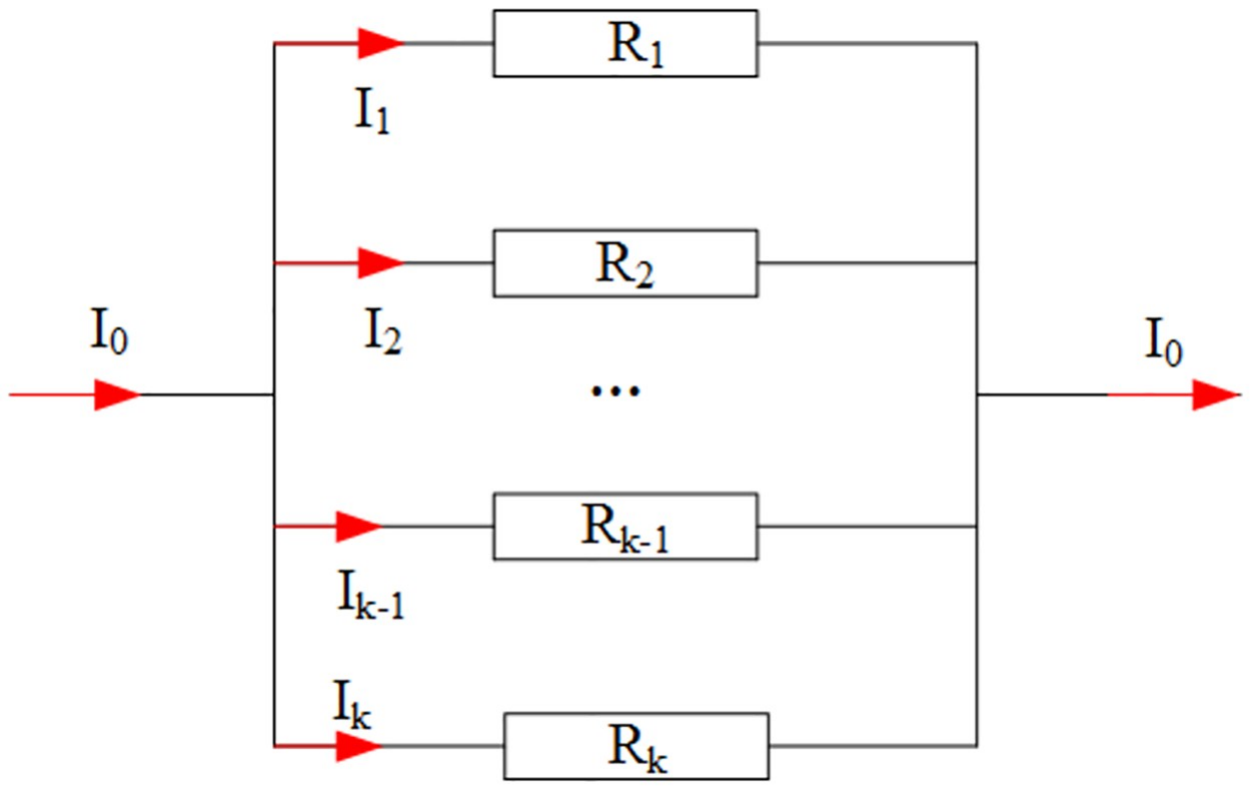
Schematic diagram of a parallel circuit.

To load the loop source with a complex shape, the loop source can be regarded as a combination of a series of electric dipoles (EDs), as shown in Fig 3A, each of which may have its own direction. Subsequently, the current density of each ED can be described as follows:

$$J_{s}=Ids\left(\begin{array}{c} lx\delta \left(x-x_{x}\right)\delta \left(y-y_{x}\right)\delta \left(z-z_{x}\right)x+\\ ly\delta \left(x-x_{y}\right)\delta \left(y-y_{y}\right)\delta \left(z-z_{y}\right)y+lz\delta \left(x-x_{z}\right)\delta \left(y-y_{z}\right)\delta \left(z-z_{z}\right)z \end{array}\right),$$ (4)

where *I* is the source current, *ds* is the ED length, *lx*, *ly*, and *lz* are the lengths of the EDs in three directions of the Cartesian coordinate system, and (*x*_*x*_, *y*_*x*_, *z*_*x*_), (*x*_*y*_, *y*_*y*_, *z*_*y*_), and (*x*_*z*_, *y*_*z*_, *z*_*z*_) are the center coordinates of the EDs, as shown in Fig 3B. *δ* denotes the delta function. We note that the transmitting source can freely intersect with the tetrahedral element and is not restricted by the grid (see Fig 4). The coil may pass through the tetrahedral element or fall onto its edges or surfaces (see Fig 5). In the latter case, the current is proportionally distributed to the adjacent cells according to Ohm’s law of parallel circuits (see Fig 6). In the case where the transmitting source falls on the unit interface, the source current is divided into two currents according to the following equation:

$$rat^{e}=\frac{\left(\sigma^{e}V^{e}\right)}{\left(\sigma^{1}V^{1}+\sigma^{2}V^{2}\right)},e=1,2,\ldots ,$$ (5)

where *σ*^*e*^ and *V*^*e*^ are the conductivity and volume of the two elements containing the interface, respectively, and *rat*^*e*^ is the proportion of current at the e-th element. When the transmitting source falls on the edge of the cell, the source current is distributed among all the cells sharing the edge according to the following equation:

$$rat^{e}=\frac{\sigma^{e}V^{e}}{\sum_{j=1}^{N}\sigma^{j}V^{j}}e=1,K,\ldots ,$$ (6)

where *K* is the number of elements containing the same edges.

The target area was discretized using a tetrahedral mesh. Adopting a vector FEM for discretizing the spatial variables of Eq (3), after finite-element analysis, applying appropriate boundary conditions to the equation, the matrix equation can be obtained as follows:

AE=b, (7)

where

$$\left\{\begin{array}{c} \begin{array}{c} A=K^{e}-i\omega \mu_{0}M_{1}^{e}\\ b=i\omega \mu_{0}\sum_{e=1}^{N_{e}}\int N_{i}^{e}\cdot J_{s}dv \end{array}\\ \begin{array}{c} K^{e}=\int_{\Omega_{e}}\left(\nabla \times N_{i}\right)\cdot \left(\nabla \times N_{j}\right)dv\\ M_{1}^{e}=\int_{\Omega_{e}}N_{i}\sigma N_{j}dv \end{array} \end{array}.\right.$$ (8)

Considering that it is not easy to converge with the iterative solver and solve the vector FEM equations, we use the parallel direct solver MUMPS and solve the finite-element Eq (8) to improve the calculation efficiency and accuracy. Subsequently, the desired magnetic field is obtained using Faraday’s law.

### SNMR principles

The physical property used in SNMR applications is the spin of hydrogen protons in water molecules. The SNMR measurement process can be briefly described as follows: for hydrogen protons in a stable geomagnetic field, the direction of their magnetic moments is consistent with the direction of the geomagnetic field. If the excitation field of the Larmor frequency is applied in the direction perpendicular to the geomagnetic field, hydrogen protons absorb the energy of the excitation field, their magnetic moments deviate from the direction of the geomagnetic field, and they enter a resonance state. When the excitation field is cancelled, hydrogen protons release the absorbed energy, and their magnetic moments gradually return to the direction of the geomagnetic field [19, 20].

To obtain a nuclear magnetic resonance (NMR) signal, the oscillating current in the underground medium must be excited with the Larmor frequency (ω_*L*_ = 2*πf*_*L*_) through the transmitting coil in a very short time. This frequency can be easily calculated according to the intrinsic local Earth’s magnetic field B0, which ranges between approximately 25 and 65 μT. The excitation magnetic field B_*T*_ is generated using the applied pulse moment. The strength and direction of this magnetic field B_*T*_ are determined by the underground resistivity structure and the excitation and reception device. The component of B_*T*_ perpendicular to B0 is ${\text{B}}_{T}^{⊥}$; at this time, ${\text{B}}_{T}^{⊥}$ interacts with the spin characteristics of hydrogen atoms, resulting in their magnetization deviating from the equilibrium state. Because the spin precession motion of a hydrogen atom has circular polarization characteristics, any elliptical polarization field can be decomposed into a pair of circularly polarized components with different directions, according to the following formula:

$${\text{B}}_{T}^{⊥}={\text{B}}_{T}^{+}+{\text{B}}_{T}^{-},$$ (9)

where the positive rotation component ${\text{B}}_{T}^{+}$ rotates clockwise, corresponding to the spin direction of the hydrogen nucleus. The anti-rotation component ${\text{B}}_{T}^{-}$ rotates counterclockwise, i.e., opposite to the spin direction of the hydrogen nucleus. The rotation axes of both are the directions of the geomagnetic field ${\hat{b}}_{0}$. In contrast to ${\text{B}}_{T}^{-}$, ${\text{B}}_{T}^{+}$ determines the deviation of the spin from the direction aligned with B0. The complex expression of ${\text{B}}_{T}^{⊥}$ in the frequency domain is as follows [19, 20]:

$${\text{B}}_{T}^{⊥}\left(r,\omega_{L}\right)=e^{i\zeta \left(r,\omega_{L}\right)}\cdot [\alpha \left(r,\omega_{L}\right)\cdot \hat{b}+i\beta \left(r,\omega_{L}\right)\cdot {\hat{b}}_{0}\times \hat{b}],$$ (10)

where *r* is a point in the 3D space, *ω*_*L*_ is the Larmor frequency, and *α* and *β* are the semi-major and semi-minor axes of the elliptical polarization components, respectively. The phase of the ellipse is *ζ*, while $\hat{b}$ and ${\hat{b}}_{0}$ are the excitation and geomagnetic field directions, respectively. We can obtain the time domain expression of two different circular polarization fields ${\text{B}}_{T}^{⊥}$ and ${\text{B}}_{T}^{-}$ according to the following formula:

$${\text{B}}_{T}^{\pm }\left(r,t\right)=\frac{1}{2}I_{0}\left(\alpha \mp \beta \right)\times \left[cos\left(\omega_{L}t-\zeta \right)\hat{b}\mp sin\left(\omega_{L}t-\zeta \right){\hat{b}}_{0}\times \hat{b}\right].$$ (11)

The magnitude and unit-direction vectors of the two opposite circular polarization fields are obtained as follows:

$$\left\{\begin{array}{c} \left|{\text{B}}_{T}^{\pm }\right|=\frac{1}{2}I_{0}\left(\alpha \mp \beta \right)\\ {\hat{b}}^{\pm }=\frac{{\text{B}}_{T}^{\pm }\left(r,t\right)}{\left|{\text{B}}_{T}^{\pm }\left(r,t\right)\right|} \end{array}\right..$$ (12)

By transmission different pulse moments, we can detect different depths. The pulse moment can be obtained by multiplying the pulse duration by the current (q = *l*τ_*p*_). Therefore, the complex SNMR signal and kernel function *K*(*q*, *r*) expressions related to water content *f*(*r*) are as follows [19, 20]:

$$\text{V}\left(q\right)=\int_{ve}f\left(r\right)\cdot K\left(q,r\right)dv$$ (13)

where

$$\begin{array}{ll} K\left(q,r\right) & =-\omega_{L}M_{0}sin\left(-\gamma_{p}\frac{q}{I_{0}}\left|{\text{B}}_{T}^{+}\left(r,\omega_{L}\right)\right|\right)\\ & \times \frac{2}{I_{0}}\left|{\text{B}}_{R}^{-}\left(r,\omega_{L}\right)\right|\cdot e^{i\left(\zeta_{T}\left(r,\omega_{L}\right)+\zeta_{R}\left(r,\omega_{L}\right)\right)}\\ & \times \left[{\hat{b}}_{R}\left(r,\omega_{L}\right)\cdot {\hat{b}}_{T}\left(r,\omega_{L}\right)+i{\hat{b}}_{0}\cdot \left({\hat{b}}_{R}\left(r,w_{L}\right)\times {\hat{b}}_{T}\left(r,\omega_{L}\right)\right)\right]. \end{array}$$ (14)

*M*_0_ is a constant, and denotes the hydrogen-proton magnetization in each cubic meter. The subscripts T and R indicate transmission and reception, respectively. As mentioned above, B_*R*_ represents the magnetic field generated by a current with amplitude equal to unity in the receiver circuit and ${\text{B}}_{R}^{-}$ is the counter-rotation component of B_*R*_. ${\hat{b}}_{R}$, ${\hat{b}}_{T}$, and ${\hat{b}}_{0}$ are the directions perpendicular to the transmission, reception, and geomagnetic fields, respectively. *ζ*_*T*_ and *ζ*_*R*_ are the phase-delay terms of the excitation field propagating from the transmission loop to position **r** and the SNMR response returning from position **r** to the reception coil, respectively. *ζ*_*T*_ (*r*, *ω*_*L*_) + *ζ*_*R*_ (*r*, *ω*_*L*_) is the initial phase caused by the resistivity; the initial phase can also be caused by the geometry of the loop as well as the off-resonance. *r* expresses the coordinates of the center points of the transmission and reception loops on the ground. $\gamma_{p}\left|{\text{B}}_{T}^{+}\left(r,\omega_{L}\right)\right|\tau_{p}=\theta_{T}\left(r,\tau_{p}\right)$ is the tip angle. The tip angle changes in the entire volume, and its value is determined by the pulse time and ${\text{B}}_{T}^{+}$ amplitude.

#### Kernel function

The SNMR measures the volume-averaged physical properties of the subsurface in a given sensitive volume. High-precision numerical calculation of the magnetic-resonance sensitivity (i.e., kernel) function is essential for forward modeling and inversion of SNMR data. In a given field setting, the changes in the kernel function reveal the impact of a certain change in the physical properties at a certain location in the SNMR data. To complete the forward modeling of the surface magnetic resonance, it is necessary to divide the detection area into 3D grids. The kernel function depends on the Earth’s magnetic field, energizing magnetic field, Larmor frequency, and pulse duration. Considering that the detection area close to the coil has a high resolution, while the detection space far away has a low resolution, the non-uniform tetrahedral mesh subdivision is selected, i.e., the size of the tetrahedron is set according to the resolution. In this way, the forward magnetic-resonance calculation efficiency and accuracy of the data are guaranteed. By solving Eq 3, the vertical component of the excitation magnetic field is obtained, as well as a 3D kernel function *K*(*q*, *r*) for each element by calculating the kernel function at all nodes and integrating each partition element.

$$K\left(q,r\right)=\int_{ve}K_{3D}\left(q,r\right)dv=ve\sum_{i=1}^{N}\frac{1}{N}K_{3D}\left(q,r_{i}\right),$$ (15)

where *ve* is the volume of element *N* = 4.

**Fig 7:**
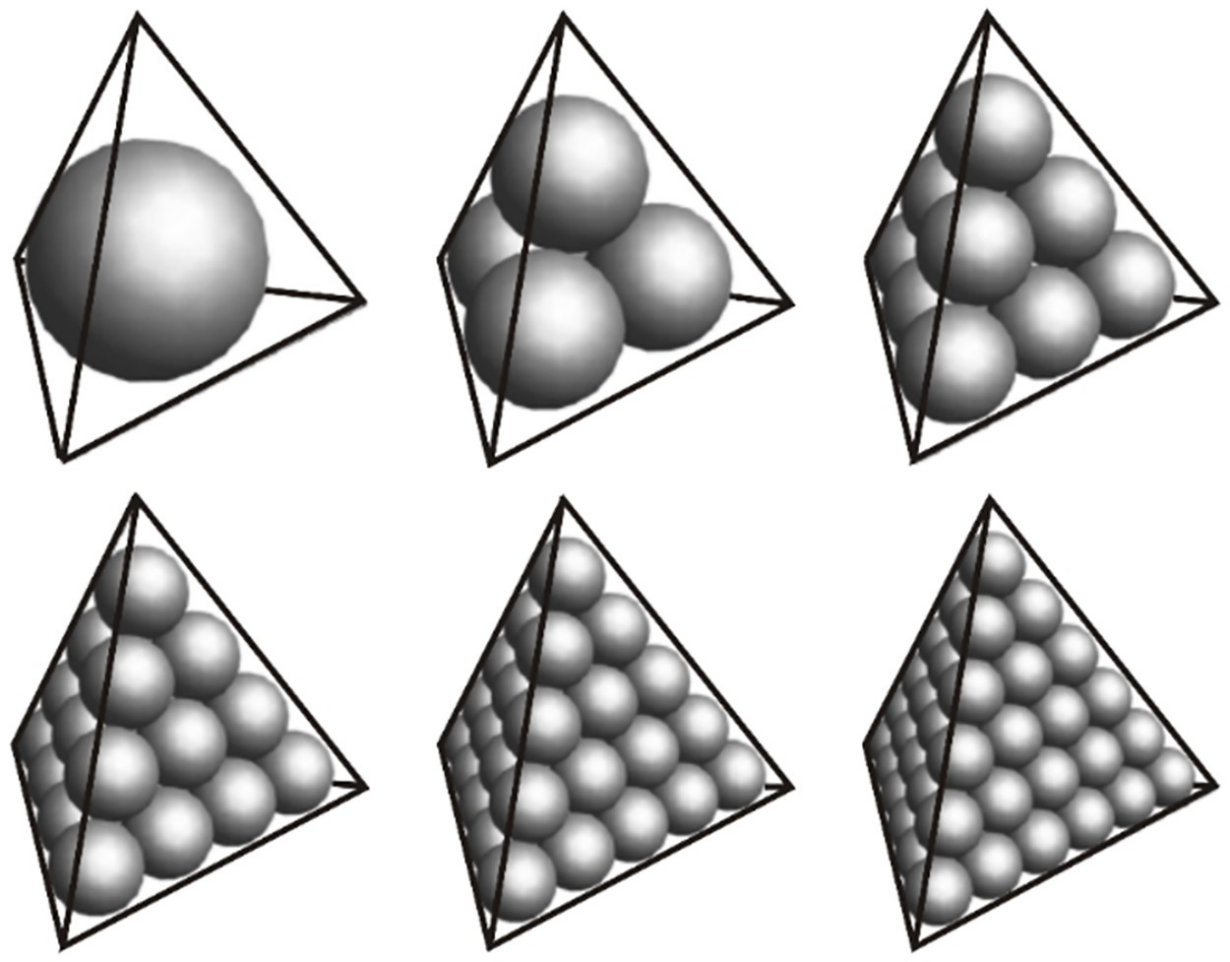
Schematic diagram of cubic close-packed structures of the element integration points.

**Fig 8:**
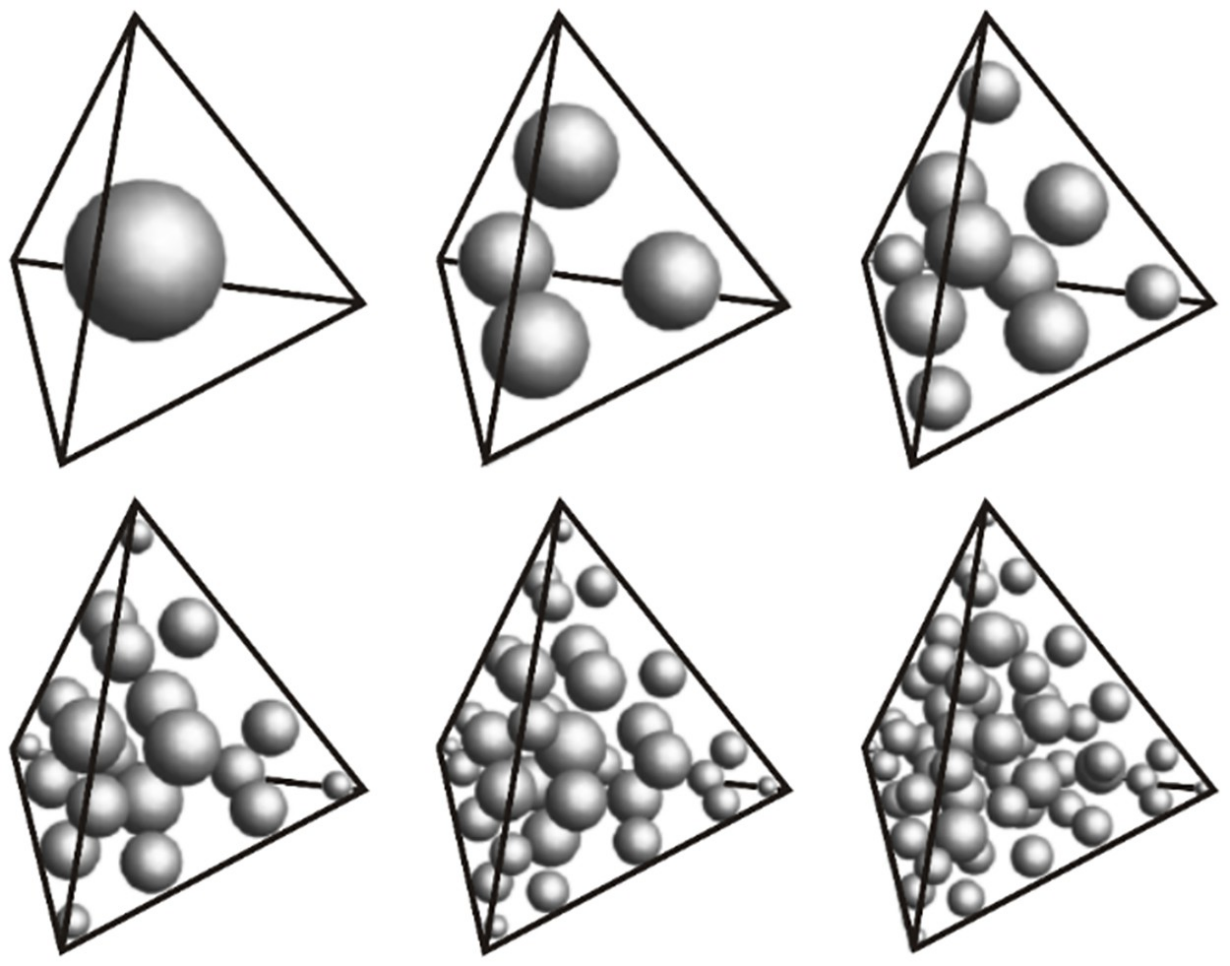
Position and weights of integral points. The size of each sphere represents the relative weight of the quadrature point. First from left to right, then from top to bottom, the integration system with order 1–6, and the total number of integration points are 1, 4, 10, 20, 35 and 56, respectively.

However, owing to the sinusoidal term in Eq (14), the kernel function has the characteristic of a rapid oscillation near the surface coil and it is required that the mesh is encrypted near the coil to compute the 3D kernel function. Nevertheless, the increase in the number of elements increases dramatically the required computational space and time. To address this problem, we adopt a symmetric quadrature rule (i.e., SQR_CCP) for calculating the kernel function of elements in the region near the coil. This quadrature rule has a cubic close-packed lattice arrangement. For a tetrahedral element, the optimized rules are shown in Fig 7, the position and weight of the interpolation points are given in Fig 8 and the values of a_*i*,*j*_ and weights are shown in the S1 Appendix. It is evident that all integration points are in tetrahedral elements. Unlike other integration systems, there are no points on the surface or edges. Table 1 provides the theoretical accuracy of the proposed integral system. The tetrahedron-element integral of the SNMR kernel function *K*_3*D*_(*q*, *r*_*i*_) can be calculated using a linear quadrature approximation:

$$K\left(q,r\right)=\int_{ve}K_{3D}\left(q,r_{i}\right)dv=ve\sum_{i=1}^{N_{p}}W_{i}K_{3D}\left(q,r_{i}\right),$$ (16)

where *W*_*i*_ is the quadrature weight, *r*_*i*_ is the point location, and *N*_*p*_ is the number of integration points. The quadrature points *r*_*i*_ can be expressed using four vertices of the tetrahedron elements *e*_1_, *e*_2_, *e*_3_, *e*_4_ as

$$r_{i}=\sum_{j=1}^{4}a_{ij}e_{j}$$ (17)

where *a*_*ij*_ is the quadrature point. The quadrature rule that is used for calculating the kernel function can be fully specified as long as the appropriate values for *a*_*ij*_ and *W*_*i*_ are determined. From Table 1, it can be seen that with an increase in the number of integration points, the accuracy of the kernel function is improved. Figs 2 and 3 show the optimized rules. In this study, we used the 35-point rule (i.e., Np = 35) for calculating the kernel function of the element. These integration points can be divided into five groups for this configuration. The first group is the “vertices” near the four corners of the tetrahedron, and the second group is the three points located on each edge, i.e., a total of 12 points called “outer-edge” points. The third group is the points near the center of each edge called “inner edge” points. The fourth group consists of three points that form a triangle, each close to each face, i.e., a total of 12 points, which we call the “face” points. The last group is the center point of the tetrahedral element, called the “interior” point (see Fig 7).

**Table 1:** Theoretical accuracy of the SQR_CCP integral system.

N_*l*_	N_*p*_	order
1	1	δ^2^
2	4	δ^3^
3	10	δ^5^
4	20	δ^6^
5	35	δ^7^
6	56	δ^9^

This SQR_CCP integral system has many notable characteristics, including the following:

1. This scheme minimizes the truncation error; therefore, in a sense, it is optimal.
2. The positions of the integral points were symmetrically distributed; the scheme has nothing to do with vertex sorting.
3. All orthogonal points are located inside the tetrahedral element.
4. The weight coefficients of integral points are non-negative.

## Model studies

To validate our 3D algorithm and evaluate how topography, loop size/shape, and subsurface conductivity impact SNMR signals, we created four synthetic studies. First, we designed a horizontally layered medium model for verifying the correctness of the algorithm while calculating the excitation magnetic field. Next, we designed an ellipsoidal water-body model and considered different buried depth conditions for proving the calculation accuracy and efficiency of our proposed method. Finally, we considered more realistic models for investigating the influence of undulating topography and conductivity characteristics on the SNMR response. The examples in this paper are all calculated on a computing platform; the parameters of the platform are one Inter(R) Core (TM) I7-6700 CPU, 3.41 GHz. The size of the memory was 64G, Windows 10 system.

### Synthetic sample 1—Horizontally layered model

**Fig 9:**
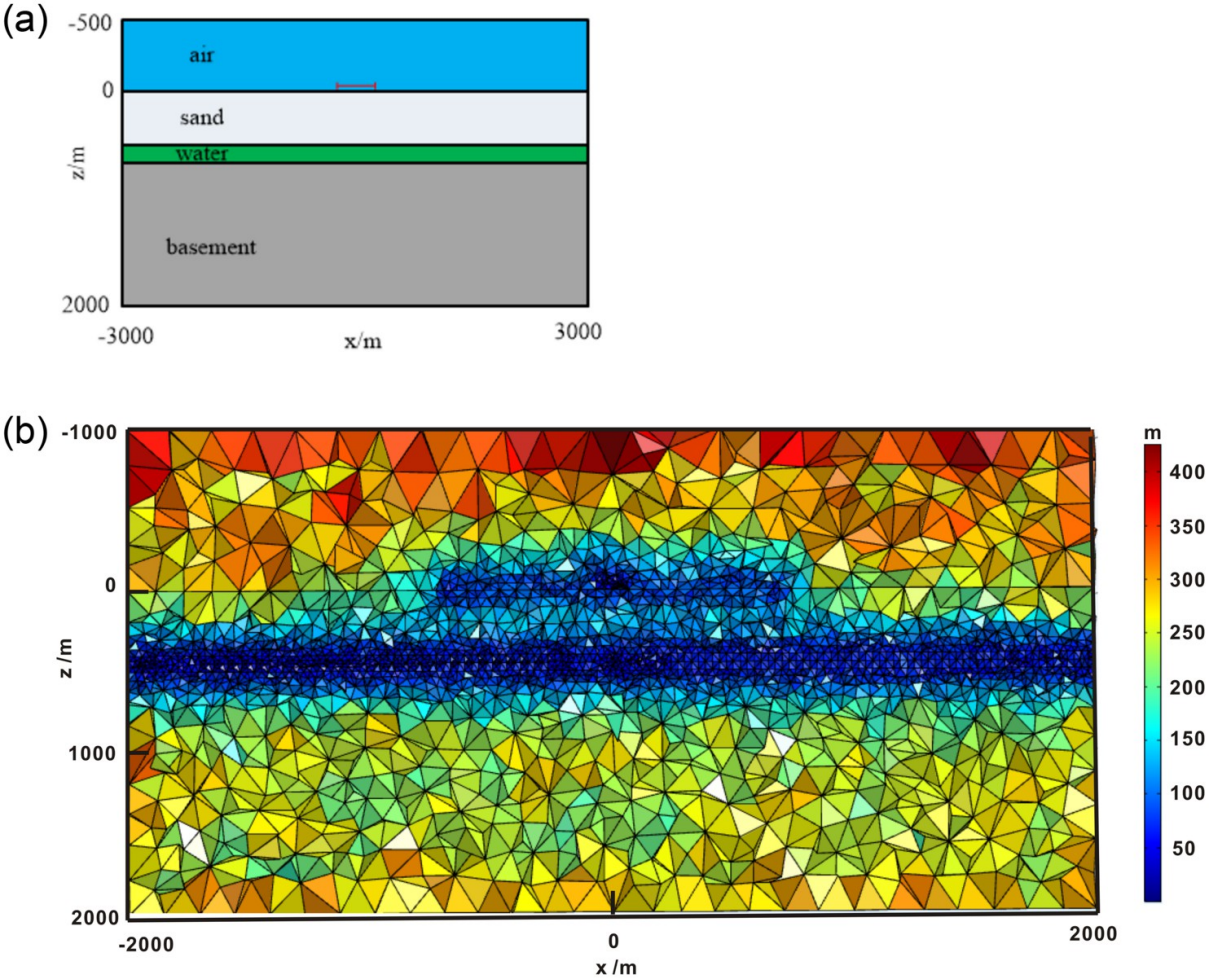
(A) Schematic diagram of a 1D layered model. (B) Vertical section of the mesh at y = 0 m.

**Fig 10:**
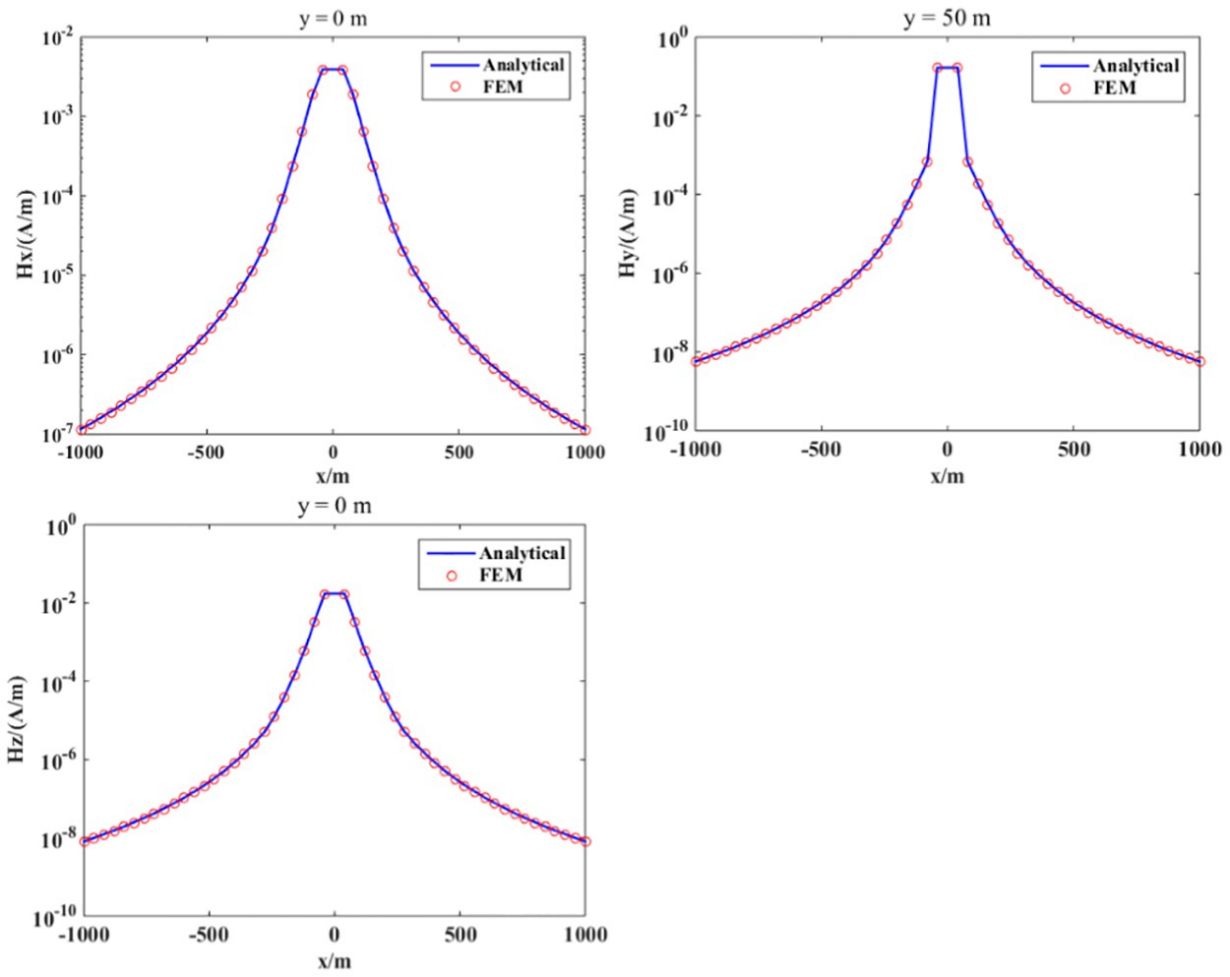
Comparison of the FEM results with the semi-analytical solution. From top to bottom are the Hx, Hy and Hz magnetic-field components.

In this section, we verify the correctness of our proposed algorithm for calculating the energizing electromagnetic field (magnetic field). First, we considered a 1D layered geoelectric model with an isotropic medium, as shown in Fig 10. The conductivity of air was 10^−6^ s/m; the secondary layer was sand with 0.1 s/m conductivity. The thin layer was water with 3 s/m conductivity. The last layer was a basement with 0.01 s/m conductivity. The depths of air, sand, and water were 1000, 400, and 200 m, respectively. A horizontal rectangular coil excited the electromagnetic field. The side length of the coil was 100 m and the current distance was 100 Am. The center of the coil was (0, 0, -1) m, z = 0 m was the surface, and the excitation frequency was 2330 Hz. The whole modeling domain was set as Ω = {-3 *km* ≤ *x*,*y* ≤ 3km; −1 km ≤ z ≤ 2 km}. We used an unstructured mesh for discretizing the computational domain. To obtain a high-precision solution, the mesh of the area close to the excitation source and the receiving station were locally refined, as shown in Fig 9. For comparison with our numerical simulation results, we adopted a Hankel integral technology for calculating the semi-analytical solution as a reference solution [21]. Fig 10 shows the comparison of the semi-analytical solution between the finite element results. The FEM solution was found to be highly consistent with a semi-analytical solution. Therefore, our forward-modeling approach for calculating the energizing electromagnetic energy was effective. The calculation results of this 1D model confirmed the accuracy and practicality of the proposed algorithm for SNMR numerical simulations.

### 3D kernel function

**Fig 11:**
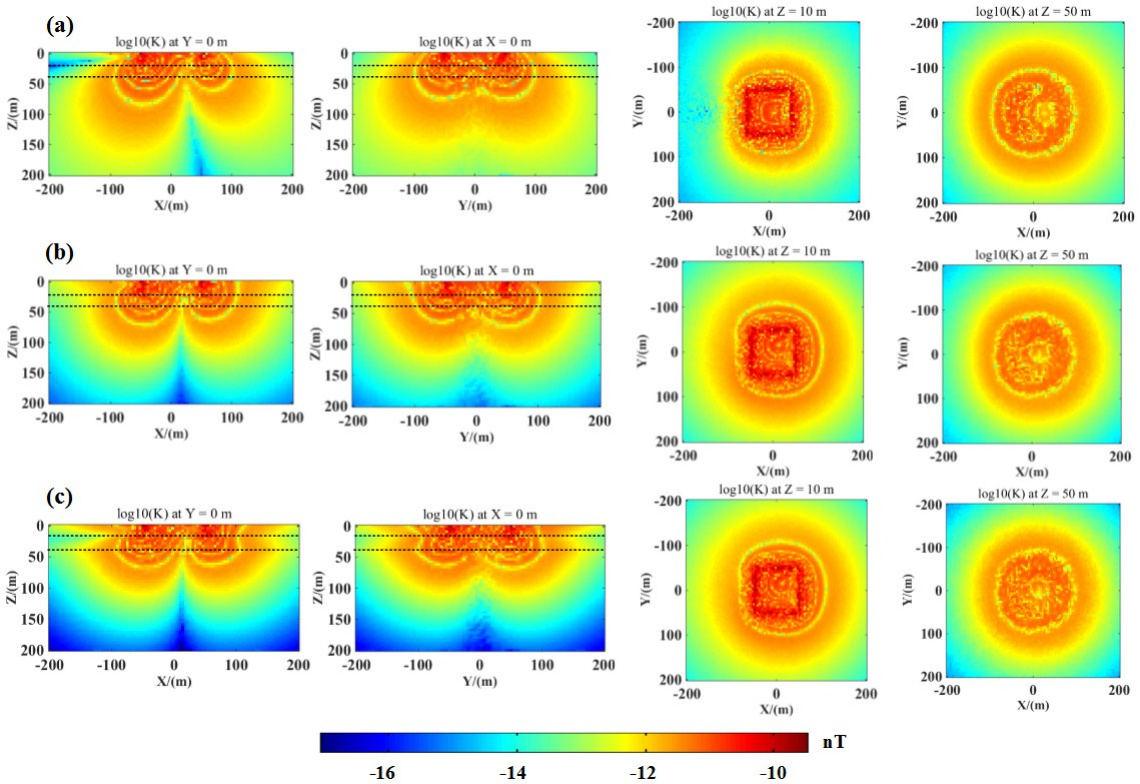
Results of the SNMR kernel functions for different typical geological models. (a) a half-space with 10000 Ωm homogeneous resistivity. (b) A half-space with 20 Ωm homogeneous resistivity. (c) A horizontally layered medium model with conductivities of 10, 100, and 10 Ωm and 20 m thickness. The subgraphs in the first, second, third, and fourth columns are slices of the 3D SNMR kernel functions at y = 0 m, x = 0 m, z = 10 m, and z = 50 m, respectively. The pulse moment of this example was 10 As.

To observe the change law of the 3D kernel function more intuitively, Fig 11 shows the result of the 3D modeling of the SNMR kernel function. We determined the SNMR kernel functions for three geological models: (a) a homogenous 10000 half-space, (b) a homogenous 20 Ωm half-space and (c) a horizontally layered medium model with a three-layer structure, with 10, 100, 10 Ω resistivities, and 20 m thickness. The Larmor frequency was 2330 Hz. The Earth’s magnetic field intensity was 50, 030 nT, while the inclination and declination were 70 and 0°. The pulse moment was 10 A. We adopted a coincident rectangular loop with a size of 100 × 100 m for exciting the electromagnetic and reception signals. The center coordinates of the loop were (0, 0, 0). The x and y directions were aligned to the east–west and north–south directions, respectively.

In Fig 11A and 11B, it is evident from the rows 1 and 2 that higher conductivity of the underground medium resulted in shallower penetration depth of the 3D SNMR kernel function. Moreover, there was an obvious asymmetric distribution in the x = 0 slice of the 3D SNMR kernel function (see columns 1, 3, and 4). In addition, the 3D kernel function was basically symmetrically distributed with respect to x = 0 (see column 2), and the sensitivity near the coil maximum gradually oscillated with increasing distance and depth. Compared with the homogeneous half-space model, the 3D SNMR kernel function of the horizontally layered medium model (row 3) had sharp structural features at the x = 0 m section diagram. Moreover, the kernel distribution was markedly different from the half-space model. In columns 3 and 4, in the shallow areas of the subsurface, the 3D SNMR kernel function had a square-shaped pattern. However, in deep subsurface areas, the 3D surface NMR kernel function had a circular pattern. This was caused by the shape of the coil. In shallow regions, according to the law of electromagnetic-field propagation, the energizing electromagnetic field generated by a rectangular coil is markedly different from a circular loop with a similar size. As the depth increases, this difference decreases; therefore, in the deep areas, the electromagnetic fields excited by rectangular and circular loops will look the same.

### Comparison of results before and after the improvement of the integral scheme

**Fig 12:**
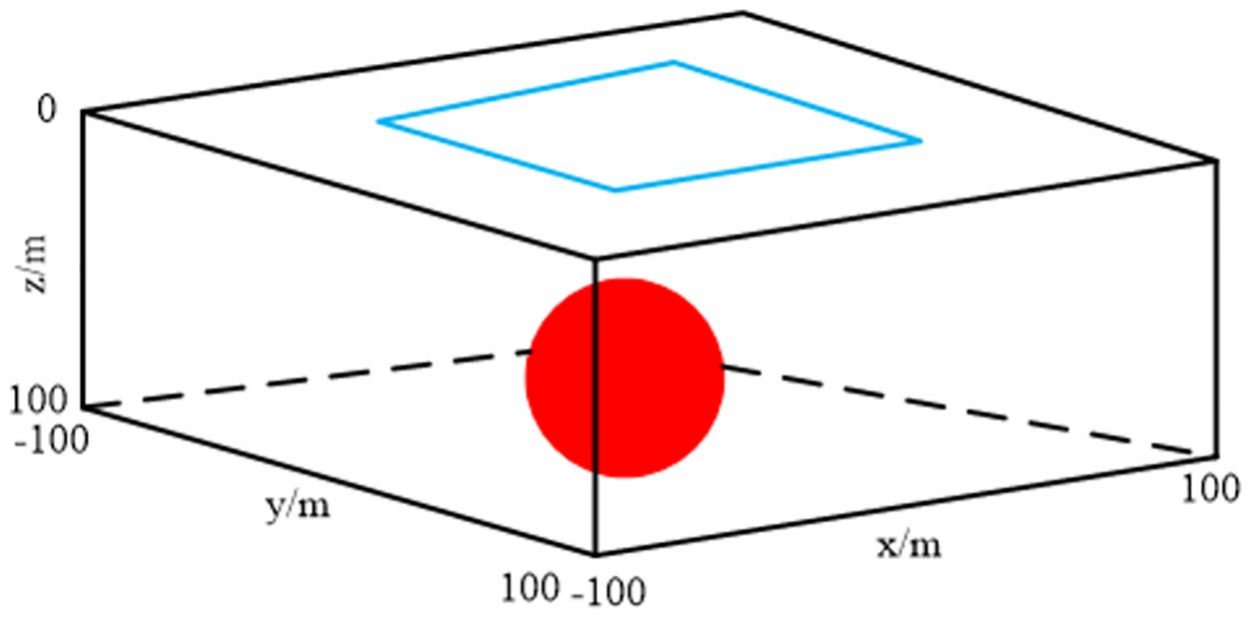
Deep single water-body model. The red sphere is the water-bearing object, while the blue rectangle is the transmission loop.

**Fig 13:**
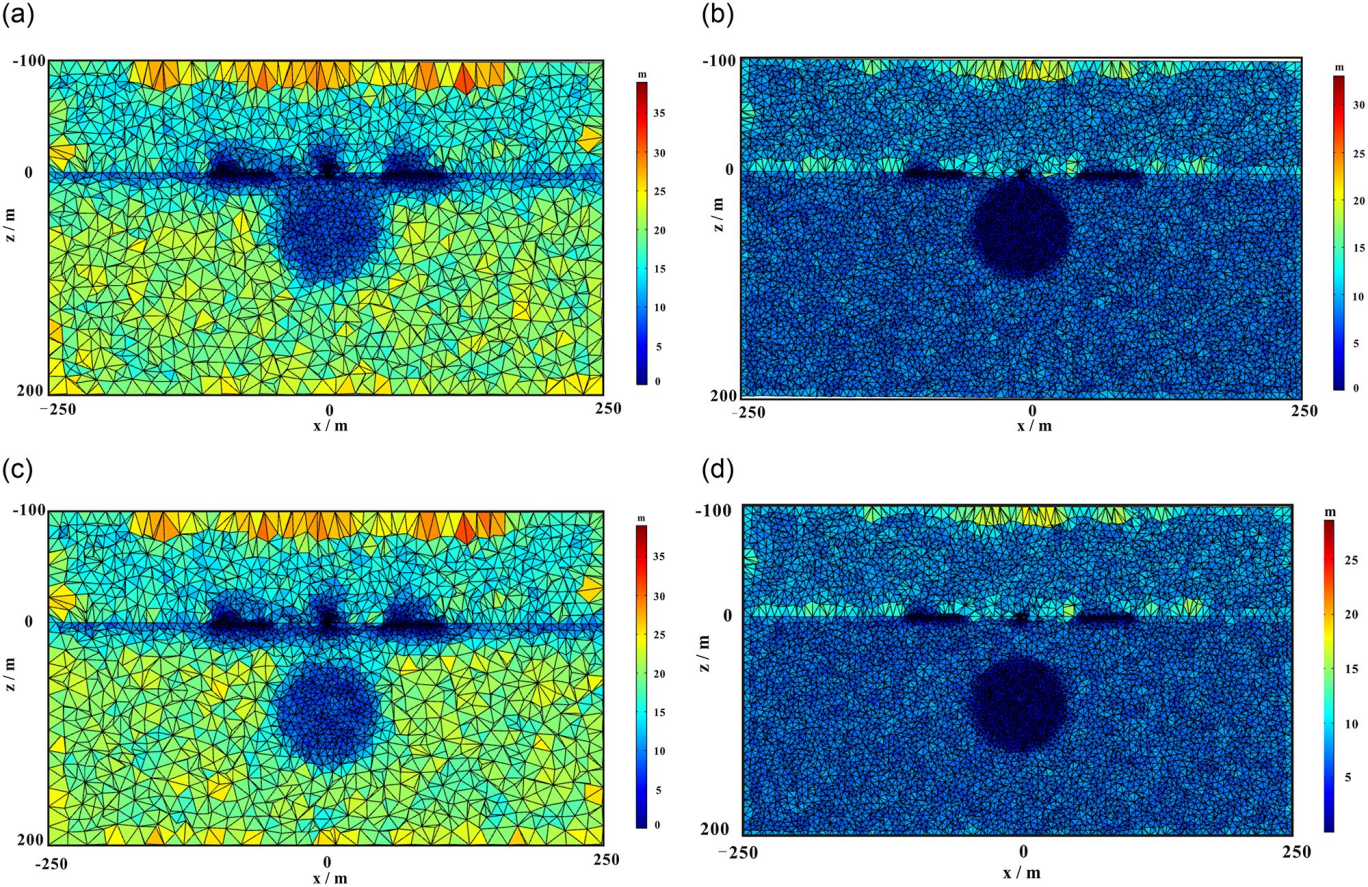
Vertical section at y = 0 of (A) a coarse and (B) a fine tetrahedron mesh for a shallow single water-body geoelectric model. Vertical section at y = 0 of (C) a coarse and (D) a fine tetrahedron mesh for a deep single water-body geoelectric model.

**Fig 14:**
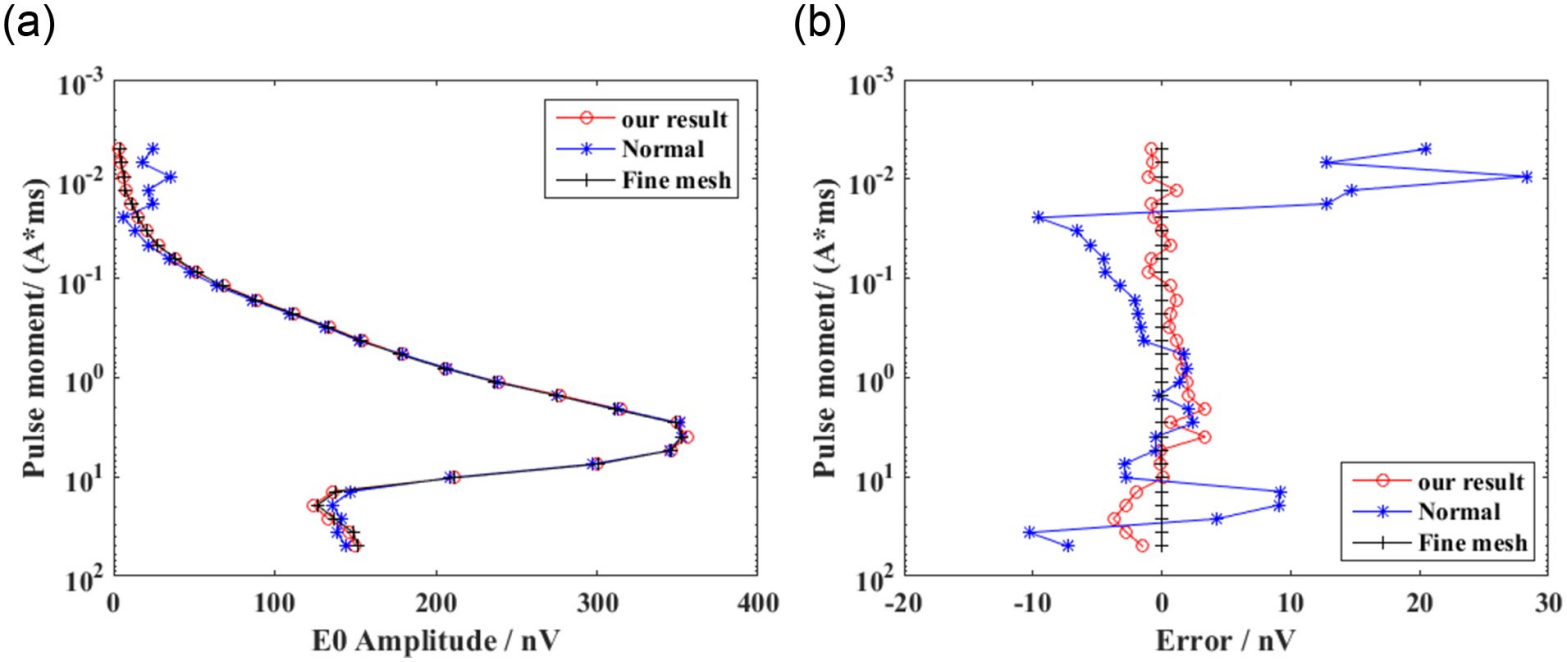
Forward calculation results with one shallow water-bearing object. (A) Amplitude and (B) error of the FID signal.

**Fig 15:**
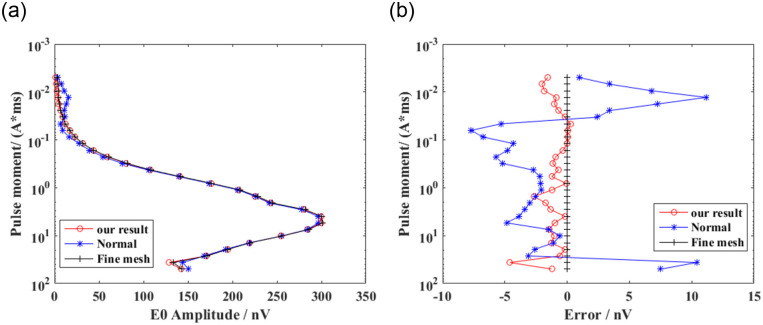
Forward calculation results with one deep water-bearing object. (A) Amplitude and (B) error of the FID signal.

To validate the effectiveness of the presented integration technology, we considered a homogeneous model with a spherical water body at different depths, as shown in Fig 12. The radius of the sphere was 40 m. The coordinates of the center of the shallow- and deep-water spheres were (0, 0, 50) and (0, 0, 80) m, respectively. The conductivities of the background medium and water were 0.01 and 0.1 s/m, respectively. The water saturation of the background medium and spherical bodies of water were 0.1 and 1.0, respectively. We placed a horizontal rectangular coil at 2 m above the ground to excite the electromagnetic field. The pulse moments ranged from 0.005 to 50 As. The Larmor frequency was 2330 Hz. The strength of the static magnetic field was 48000 nT. The inclination and declination were 60 and 0°, respectively. The computation domain was selected as Ω = {−250*m* ≤ *x*,*y* ≤ 250*m*; −100*m* ≤ *z* ≤ 200*m*}. We used an unstructured tetrahedron for discretizing the computation domain, as shown in Fig 13. For comparison, we discretized the two models with coarse and fine meshes. Similar to model 1, the unstructured mesh was locally refined near the loop source, target, and receiving station. Because there is no analytical solution for the 3D models of heterogeneous media, and considering that the fine-mesh result had higher precision, we adopted the fine-mesh result as a reference solution that is closer to the real solution for verifying the effectiveness of the new integration technology. We calculated the free induction decay (FID) signal using coarse and fine meshes. The coarse-mesh results with the new integration technology and conventional technical methods [16] are shown in Figs 14 and 15. It is evident that the solution calculated using the new integration technology was more consistent with that of the fine mesh, and its accuracy was higher than that of the conventional technical solution under the same mesh. In addition, a comparison between Figs 14A and 15A showed that the pulse moment of the maximum FID signal of the shallow water-bearing object model was smaller than that of the deep water-bearing object model. Greater water-body depths required larger pulse moments. Table 2 shows a comparison of the computation times of the two integration technologies. It is evident that the average error of the conventional technology was approximately six times that of the new integration technology under the same coarse mesh. The computation time of the new integration technology was approximately twice that of conventional techniques. The computation time of the fine mesh was approximately 10 times that of the conventional technology, but 5 times that of the new integration technology. We considered simulated results of the fine mesh as the optimal results and used them for comparison with the other two meshing methods. Thus, we concluded that the accuracy of the 3D SNMR forward modeling with the new integration method was higher than that of the conventional integration method, and the calculation efficiency was higher than the refinement of the mesh.

**Table 2:** Computational time and space analysis.

Models	Method	Elements	Integration points	Time (s)	MAX (Error) (nV)	AVG (Error) (nV)
**Shallow model**	Normal	454761	1819044	95	28.350	6.218
	Fine mesh	2068449	8273796	1016	-	-
	SCQ-CCP	454761	6821415	179	3.750	1.020
**Deep model**	Normal	473937	1895748	103	12.154	5.290
	Fine mesh	2178028	8712112	1056	-	-
	SCQ-CCP	473937	7109055	208	4.610	0.860

### Effect of topography and conductivity

**Fig 16:**
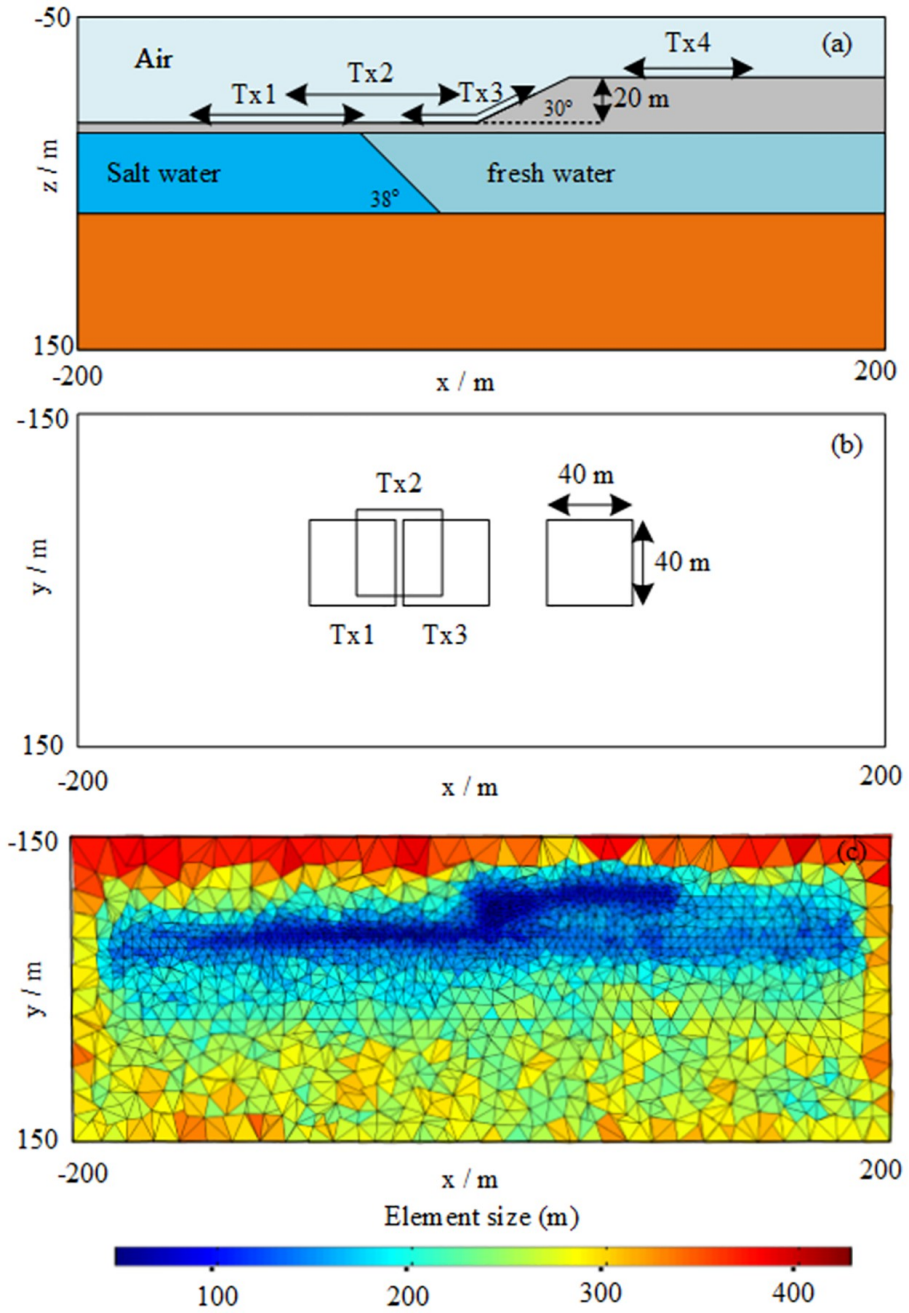
(A) Geometric diagram of the cross-section of the undulating terrain model. (B) Horizontal projection position of loops (i.e., Tx1–Tx4). (C) The unstructured meshes for the model.

To research the influence of topography and conductive structure on the SNMR response, we considered a marine-sediment model near the coast with topography as shown in the Fig 16A. The surface topography was an oblique step with 30° angle. Fig 16B shows the locations of the transmission loops Tx1–Tx4. The first layer was sand, the secondary layer was an intermediate aquifer, and there was a change in the conductivity properties in the lateral direction due to a change in the water medium from salt to fresh water. The final layer was a basement. The geometric and physical parameters of this model are listed in Table 3. The unstructured meshes for the models are shown in Fig 16C. We refined the elements of the region near the transmission source and topography. The pulse moments q were 1.60, 2.96, and 6.50 As. The Larmor frequency was 2000 Hz. The strength of the static magnetic field of the earth was 46977 nT. The inclination and declination were 70.3 and -6.4°, respectively. The side length of the rectangular loop was 40 m. First, we applied the new integration technology to calculate the 3D SNMR kernel function of coincident transmitters and receivers for the marine-sediment models with topography and examine the effect of undulating surface topography and conductivity structure. We also computed the 3D SNMR FID synthetic sounding response of different loop positions (i.e., Tx1–Tx4) by using 30 pulse moments, which ranged from 0.01 to 100 As with logarithmic interval. We adopted SNMR measurements with coincident Rx/Tx.

**Table 3:** The geometric and physical parameters of the marine-sediment model.

Medium	Depth (m)	Conductivity (s/m)	Water saturation (%)
**Air**	50	10^−6^	0
**Sand**	5	0.005	0
**Salt water**	30	3.000	100
**Fresh water**	30	0.100	30
**Basement**	-	0.010	0

**Fig 17:**
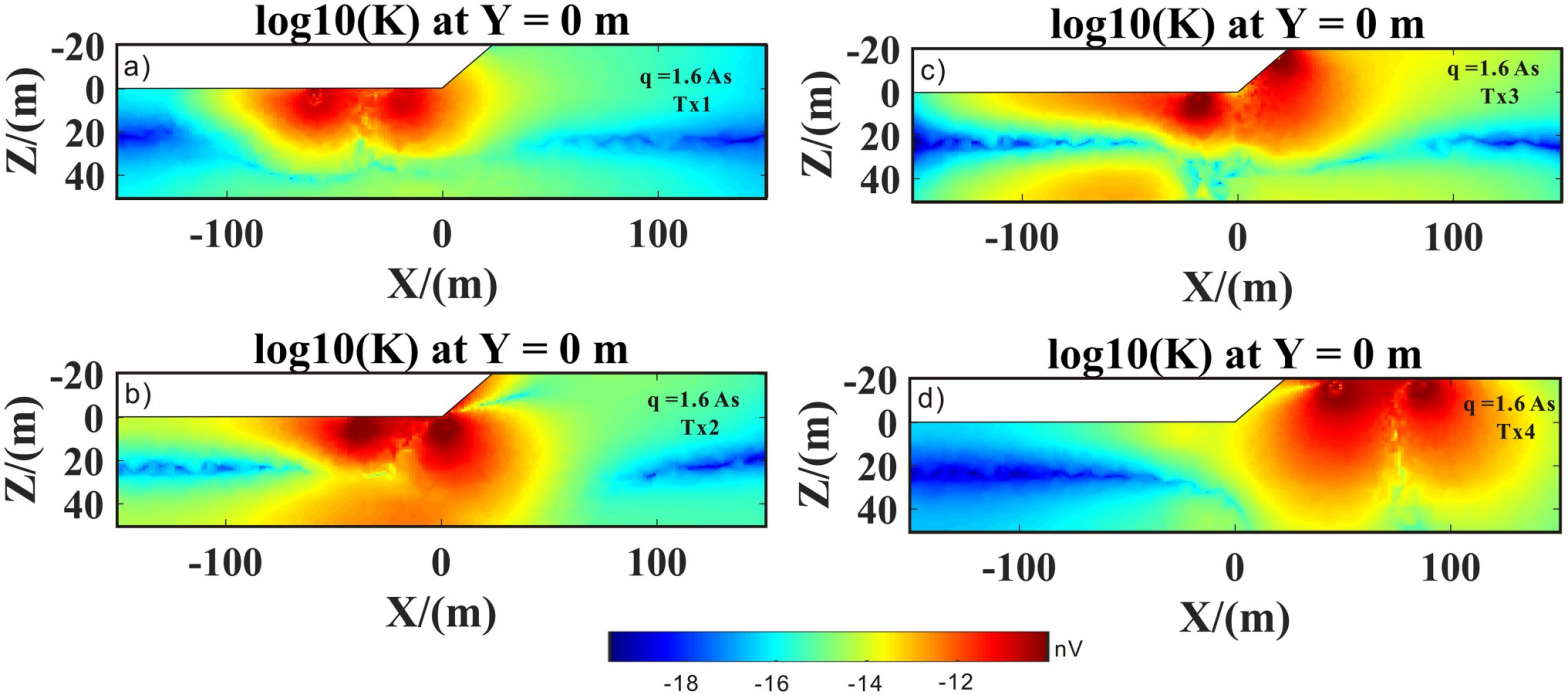
3D SNMR kernel function of the Tx1–Tx4 transmit coils, with 1.6 As pulse moment.

**Fig 18:**
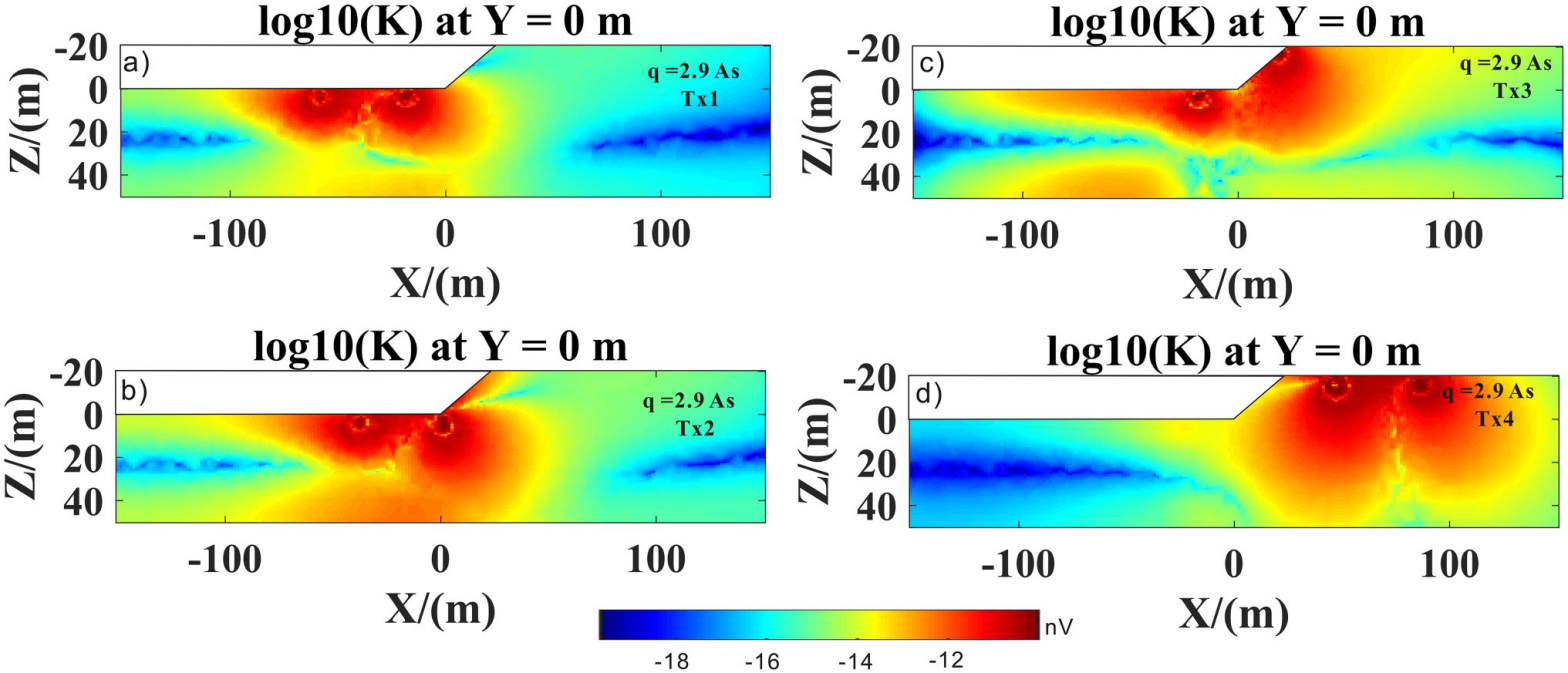
3D SNMR kernel function of the Tx1–Tx4 transmit coils, with 2.9 As pulse moment.

**Fig 19:**
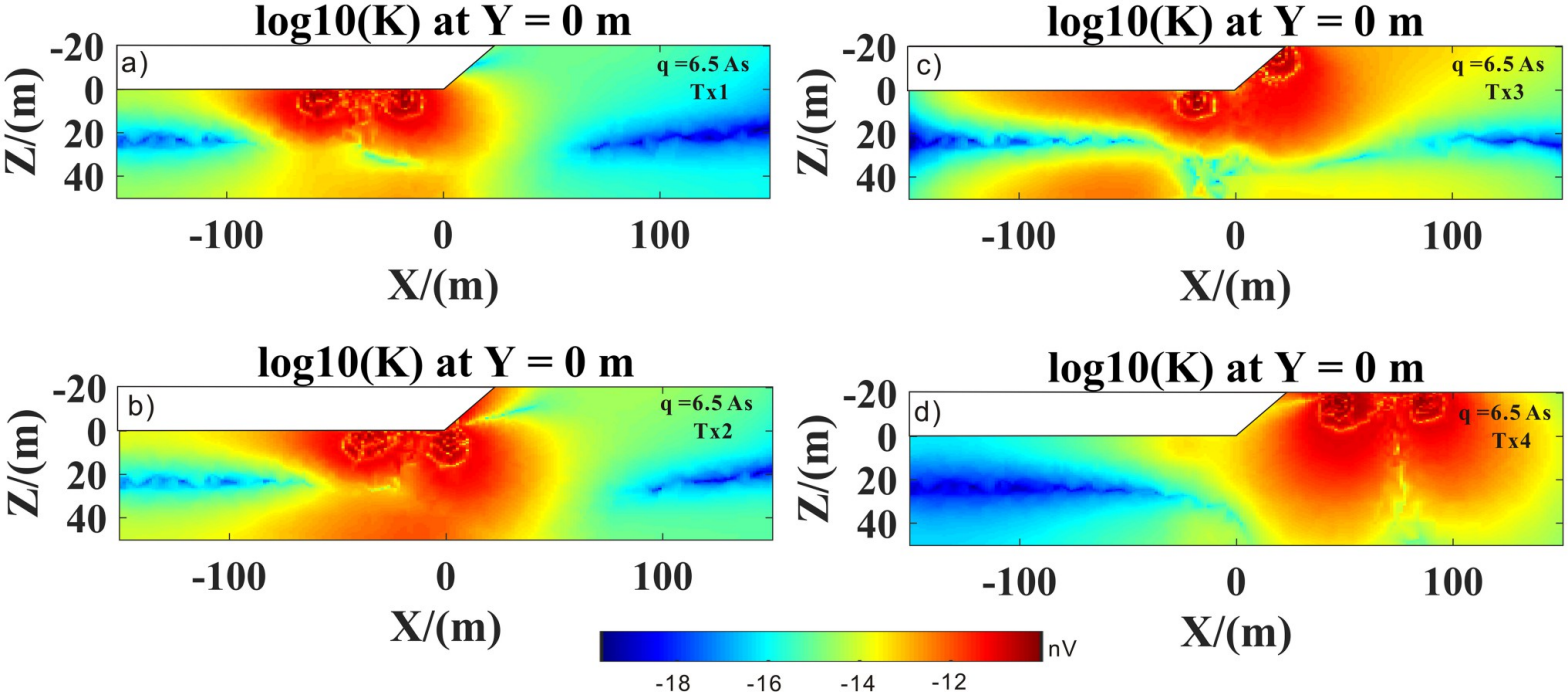
3D SNMR kernel function of the Tx1–Tx4 transmit coils, with 6.5 As pulse moment.

Figs 17 and 18 show the kernel functions of the Tx1–Tx4 loop positions for different pulse moments. Compared to the Tx4 loop position (Figs 17D, 18D and 19D), at Tx1 loop position (Figs 17A, 18A and 19A), the kernel function was significantly reduced because the magnetic field in a highly conductive medium (i.e., salt water) decayed quickly. It is also evident that penetration depth was limited. In addition, the sensitive area and depth of the kernel function of the high-resistance medium were significantly larger than those of the low-resistance medium (i.e., highly conductive medium). In Figs 17B, 18B and 19B, it is evident that the right part of the sensitive area of the kernel function was larger than the left part, which was mainly due to the conversion of the medium from a highly to a poorly conductive medium; in other words, it was obviously affected by the lateral change in the conductivity structure (i.e., transition zone). It was also affected by surface topography. In Figs 17C, 18C and 19C, it is evident that the effects of undulating surface topography and uneven distribution of conductivity structure on the kernel function were more pronounced. Therefore, we concluded that surface topography and conductive media with conductivity greater than 0.01 s/m would have a strong influence on the kernel function.

**Fig 20:**
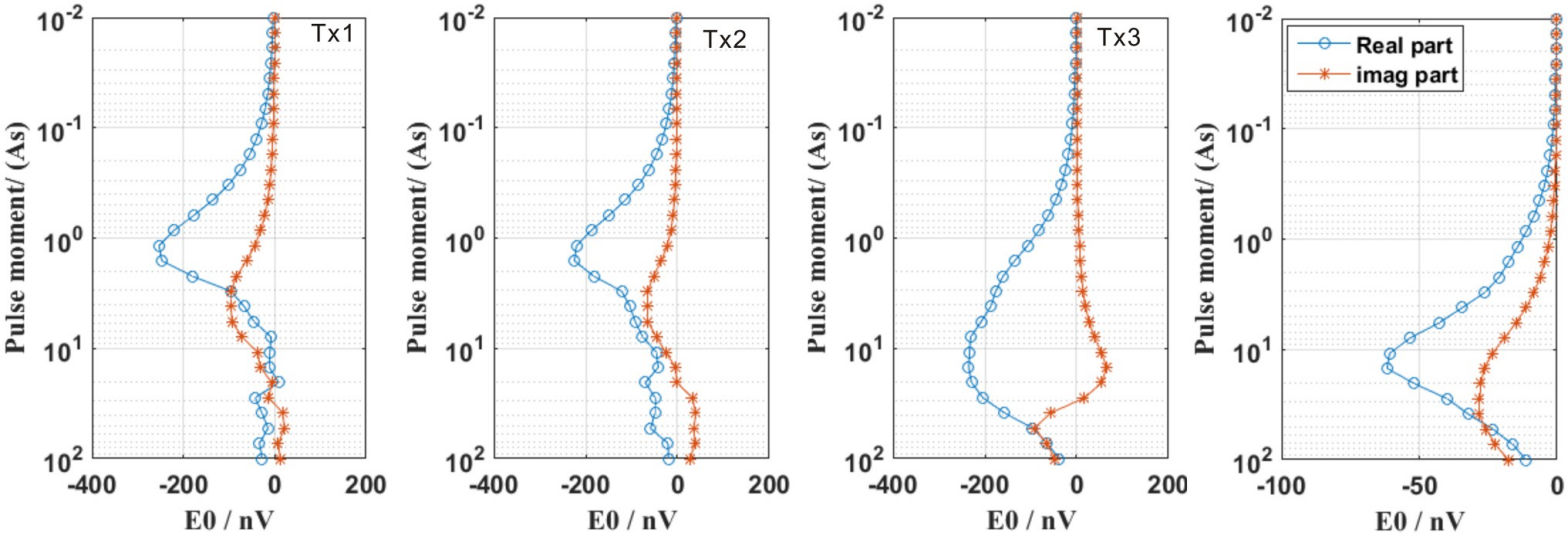
Synthetic FID sounding curves for different loop positions (i.e., Tx1–Tx4).

Fig 20 shows the SNMR responses of different pulse moments for the four reception loops Tx1–Tx4. At the Tx1 loop position, we found that the imaginary part of the FID signal of TX1 maximized at a low pulse moment (i.e., q = 4.17 As). The maximum the curve of the real part of the SNMR FID response appeared at a fairly small pulse moment (i.e., q = 1.17 As). These phenomena indicated that the loop was close to the aquifer-water layer (i.e., salt water), which was consistent with the true model. Compared with the Tx1 loop, the pulse moments for the maxima of the real and imaginary parts of the FID signal of the Tx4 loop were greater than those of the Tx1 loop and were equal to 14.87 and 38.56 As, respectively. This showed that the aquifer (i.e., fresh water) was very far from the loop (see Fig 16). Regarding the Tx2 loop, the maximum FID signal was smaller than that of the Tx1 loop, owing to the effect of lateral change in conductivity. Regarding the Tx3 loop, it is evident that the imaginary part of the FID signal switched signs when compared with those of the Tx1, Tx2, and Tx4 loops; this was caused by an increase in the distance from a highly conductive medium (i.e., salt water). In addition, it also affected the maximum of the FID signal (i.e., real and imaginary parts) at high pulse moments because the conductive medium was far from the Tx3 loop. In addition, the loop shape (which was not a standard rectangular loop) and surface topography also affected the response of the SNMR FID of the Tx3 loop. For the Tx4 loop, the maximum real and imaginary FID signals occurred at high pulse moments (i.e., 14.87 and 28.07 As, respectively) because the freshwater aquifer was far away from the position of the receiving coil. Overall, we know that the surface topography and electrical conductive medium can significantly affect the SNMR signal. If these factors are not considered in the 3D SNMR numerical simulation problem, we will likely obtain incorrect information in the inversion of SNMR data.

## Conclusion

We developed a high-precision algorithm for solving the forward-modeling problem of the surface nuclear magnetic resonance (SNMR) under the complex conditions of undulating terrain and unevenly distributed 3D conductive structures. This algorithm involved the discretization of the transmitter into the dipoles for loading the arbitrarily complex-shaped loop sources. The modeling domain was discretized using an unstructured tetrahedral mesh, and it was possible to simulate arbitrary, complex, and highly discontinuous aqueous or non-aqueous conductivity structures. We adopted a new integration algorithm based on a symmetric quadrature formula to compute the kernel functions of the elements. This rule had a remarkable feature, i.e., its basic structure was arranged according to the closely arranged lattice, which helped to effectively maintain the inherent symmetry of any tetrahedral element. The forward-modeling results of two 3D water-bearing models showed that the accuracy of the new integration algorithm was higher than that of conventional techniques under the same conditions. Although the required computational time doubled, the absolute error was reduced by six times. Compared to the refinement grid, the calculation efficiency was approximately five times higher. In summary, compared to the conventional technique, our method significantly improves the accuracy of the kernel function without increasing the computation time. In addition, we designed a typical 3D geological model to calculate the 3D SNMR response using the developed algorithm. We found that the topography and conductivity structure had strong influences on the SNMR response, which needs to be considered when interpreting field SNMR data. In the future, we will focus on the implementation of the 3D inversion SNMR data by using the developed algorithm and further verify its efficiency and accuracy.

## Supporting information

S1 Data (RAR)

S1 Appendix (DOCX)
