# Supplementary material for: A high precision finite-element forward solver for surface nuclear magnetic resonance incorporating conductivity changes and surface-topography variations
Source: PLoS One. 2022 Mar 17;17(3):e0264235. doi: 10.1371/journal.pone.0264235 (PMC8929630; doi:10.1371/journal.pone.0264235)
Supplement: S1 Appendix — (DOCX) [file pone.0264235.s002.docx]

S1 Appendix

| $N_{i}$ | point $i$ | $a_{i,1}$ | $a_{i,2}$ | $a_{i,3}$ | $a_{i,4}$ | $W_{i}$ |
| --- | --- | --- | --- | --- | --- | --- |
| 1 | 1 | 0.25000000 | 0.25000000 | 0.25000000 | 0.25000000 | 1 |
|  | | | | | | |
| 2 | 1 | 0.5854101966249680 | 0.1381966011250110 | 0.1381966011250110 | 0.1381966011250110 | 0.2500000000000000 |
|  | 2 | 0.1381966011250110 | 0.5854101966249680 | 0.1381966011250110 | 0.1381966011250110 | 0.2500000000000000 |
|  | 3 | 0.1381966011250110 | 0.1381966011250110 | 0.5854101966249680 | 0.1381966011250110 | 0.2500000000000000 |
|  | 4 | 0.1381966011250110 | 0.1381966011250110 | 0.1381966011250110 | 0.5854101966249680 | 0.2500000000000000 |
|  | | | | | | |
| 3 | 1 | 0.7784952948213300 | 0.0738349017262234 | 0.0738349017262234 | 0.0738349017262234 | 0.0476331348432089 |
|  | 2 | 0.0738349017262234 | 0.7784952948213300 | 0.0738349017262234 | 0.0738349017262234 | 0.0476331348432089 |
|  | 3 | 0.0738349017262234 | 0.0738349017262234 | 0.7784952948213300 | 0.0738349017262234 | 0.0476331348432089 |
|  | 4 | 0.0738349017262234 | 0.0738349017262234 | 0.0738349017262234 | 0.7784952948213300 | 0.0476331348432089 |
|  | 5 | 0.4062443438840510 | 0.4062443438840510 | 0.0937556561159491 | 0.0937556561159491 | 0.1349112434378610 |
|  | 6 | 0.4062443438840510 | 0.0937556561159491 | 0.4062443438840510 | 0.0937556561159491 | 0.1349112434378610 |
|  | 7 | 0.4062443438840510 | 0.0937556561159491 | 0.0937556561159491 | 0.4062443438840510 | 0.1349112434378610 |
|  | 8 | 0.0937556561159491 | 0.4062443438840510 | 0.4062443438840510 | 0.0937556561159491 | 0.1349112434378610 |
|  | 9 | 0.0937556561159491 | 0.4062443438840510 | 0.0937556561159491 | 0.4062443438840510 | 0.1349112434378610 |
|  | 10 | 0.0937556561159491 | 0.0937556561159491 | 0.4062443438840510 | 0.4062443438840510 | 0.1349112434378610 |
|  | | | | | | |
| 4 | 1 | 0.9029422158182680 | 0.0323525947272439 | 0.0323525947272439 | 0.0323525947272439 | 0.0070670747944695 |
|  | 2 | 0.0323525947272439 | 0.9029422158182680 | 0.0323525947272439 | 0.0323525947272439 | 0.0070670747944695 |
|  | 3 | 0.0323525947272439 | 0.0323525947272439 | 0.9029422158182680 | 0.0323525947272439 | 0.0070670747944695 |
|  | 4 | 0.0323525947272439 | 0.0323525947272439 | 0.0323525947272439 | 0.9029422158182680 | 0.0070670747944695 |
|  | 5 | 0.2626825838877790 | 0.6165965330619370 | 0.0603604415251421 | 0.0603604415251421 | 0.0469986689718877 |
|  | 6 | 0.6165965330619370 | 0.2626825838877790 | 0.0603604415251421 | 0.0603604415251421 | 0.0469986689718877 |
|  | 7 | 0.2626825838877790 | 0.0603604415251421 | 0.6165965330619370 | 0.0603604415251421 | 0.0469986689718877 |
|  | 8 | 0.6165965330619370 | 0.0603604415251421 | 0.2626825838877790 | 0.0603604415251421 | 0.0469986689718877 |
|  | 9 | 0.2626825838877790 | 0.0603604415251421 | 0.0603604415251421 | 0.6165965330619370 | 0.0469986689718877 |
|  | 10 | 0.6165965330619370 | 0.0603604415251421 | 0.0603604415251421 | 0.2626825838877790 | 0.0469986689718877 |
|  | 11 | 0.0603604415251421 | 0.2626825838877790 | 0.6165965330619370 | 0.0603604415251421 | 0.0469986689718877 |
|  | 12 | 0.0603604415251421 | 0.2626825838877790 | 0.2626825838877790 | 0.0603604415251421 | 0.0469986689718877 |
|  | 13 | 0.0603604415251421 | 0.2626825838877790 | 0.0603604415251421 | 0.6165965330619370 | 0.0469986689718877 |
|  | 14 | 0.0603604415251421 | 0.6165965330619370 | 0.0603604415251421 | 0.2626825838877790 | 0.0469986689718877 |
|  | 15 | 0.0603604415251421 | 0.0603604415251421 | 0.2626825838877790 | 0.6165965330619370 | 0.0469986689718877 |
|  | 16 | 0.0603604415251421 | 0.0603604415251421 | 0.6165965330619370 | 0.2626825838877790 | 0.0469986689718877 |
|  | 17 | 0.3097693042728620 | 0.3097693042728620 | 0.3097693042728620 | 0.0706920871814129 | 0.1019369182898680 |
|  | 18 | 0.3097693042728620 | 0.3097693042728620 | 0.0706920871814129 | 0.3097693042728620 | 0.1019369182898680 |
|  | 19 | 0.3097693042728620 | 0.0706920871814129 | 0.3097693042728620 | 0.3097693042728620 | 0.1019369182898680 |
|  | 20 | 0.0706920871814129 | 0.3097693042728620 | 0.3097693042728620 | 0.3097693042728620 | 0.1019369182898680 |
|  | | | | | | |
| 5 | 1 | 0.9197896733368800 | 0.0267367755543735 | 0.0267367755543735 | 0.0267367755543735 | 0.0021900463965388 |
|  | 2 | 0.0267367755543735 | 0.9197896733368800 | 0.0267367755543735 | 0.0267367755543735 | 0.0021900463965388 |
|  | 3 | 0.0267367755543735 | 0.0267367755543735 | 0.9197896733368800 | 0.0267367755543735 | 0.0021900463965388 |
|  | 4 | 0.0267367755543735 | 0.0267367755543735 | 0.0267367755543735 | 0.9197896733368800 | 0.0021900463965388 |
|  | 5 | 0.1740356302468940 | 0.7477598884818090 | 0.0391022406356488 | 0.0391022406356488 | 0.0143395670177665 |
|  | 6 | 0.7477598884818090 | 0.1740356302468940 | 0.0391022406356488 | 0.0391022406356488 | 0.0143395670177665 |
|  | 7 | 0.1740356302468940 | 0.0391022406356488 | 0.7477598884818090 | 0.0391022406356488 | 0.0143395670177665 |
|  | 8 | 0.7477598884818090 | 0.0391022406356488 | 0.1740356302468940 | 0.0391022406356488 | 0.0143395670177665 |
|  | 9 | 0.1740356302468940 | 0.0391022406356488 | 0.0391022406356488 | 0.7477598884818090 | 0.0143395670177665 |
|  | 10 | 0.7477598884818090 | 0.0391022406356488 | 0.0391022406356488 | 0.1740356302468940 | 0.0143395670177665 |
|  | 11 | 0.0391022406356488 | 0.1740356302468940 | 0.7477598884818090 | 0.0391022406356488 | 0.0143395670177665 |
|  | 12 | 0.0391022406356488 | 0.7477598884818090 | 0.1740356302468940 | 0.0391022406356488 | 0.0143395670177665 |
|  | 13 | 0.0391022406356488 | 0.1740356302468940 | 0.0391022406356488 | 0.7477598884818090 | 0.0143395670177665 |
|  | 14 | 0.0391022406356488 | 0.7477598884818090 | 0.0391022406356488 | 0.1740356302468940 | 0.0143395670177665 |
|  | 15 | 0.0391022406356488 | 0.0391022406356488 | 0.1740356302468940 | 0.7477598884818090 | 0.0143395670177665 |
|  | 16 | 0.0391022406356488 | 0.0391022406356488 | 0.7477598884818090 | 0.1740356302468940 | 0.0143395670177665 |
|  | 17 | 0.4547545999844830 | 0.4547545999844830 | 0.0452454000155172 | 0.0452454000155172 | 0.0250305395686746 |
|  | 18 | 0.4547545999844830 | 0.0452454000155172 | 0.4547545999844830 | 0.0452454000155172 | 0.0250305395686746 |
|  | 19 | 0.4547545999844830 | 0.0452454000155172 | 0.0452454000155172 | 0.4547545999844830 | 0.0250305395686746 |
|  | 20 | 0.0452454000155172 | 0.4547545999844830 | 0.4547545999844830 | 0.0452454000155172 | 0.0250305395686746 |
|  | 21 | 0.0452454000155172 | 0.4547545999844830 | 0.0452454000155172 | 0.4547545999844830 | 0.0250305395686746 |
|  | 22 | 0.0452454000155172 | 0.0452454000155172 | 0.4547545999844830 | 0.4547545999844830 | 0.0250305395686746 |
|  | 23 | 0.5031186450145980 | 0.2232010379623150 | 0.4547545999844830 | 0.0504792790607720 | 0.0479839333057554 |
|  | 24 | 0.2232010379623150 | 0.5031186450145980 | 0.4547545999844830 | 0.0504792790607720 | 0.0479839333057554 |
|  | 25 | 0.2232010379623150 | 0.2232010379623150 | 0.5031186450145980 | 0.0504792790607720 | 0.0479839333057554 |
|  | 26 | 0.5031186450145980 | 0.2232010379623150 | 0.0504792790607720 | 0.2232010379623150 | 0.0479839333057554 |
|  | 27 | 0.2232010379623150 | 0.5031186450145980 | 0.0504792790607720 | 0.2232010379623150 | 0.0479839333057554 |
|  | 28 | 0.2232010379623150 | 0.2232010379623150 | 0.0504792790607720 | 0.5031186450145980 | 0.0479839333057554 |
|  | 29 | 0.5031186450145980 | 0.0504792790607720 | 0.2232010379623150 | 0.2232010379623150 | 0.0479839333057554 |
|  | 30 | 0.2232010379623150 | 0.0504792790607720 | 0.5031186450145980 | 0.2232010379623150 | 0.0479839333057554 |
|  | 31 | 0.2232010379623150 | 0.0504792790607720 | 0.2232010379623150 | 0.5031186450145980 | 0.0479839333057554 |
|  | 32 | 0.0504792790607720 | 0.5031186450145980 | 0.2232010379623150 | 0.2232010379623150 | 0.0479839333057554 |
|  | 33 | 0.0504792790607720 | 0.2232010379623150 | 0.5031186450145980 | 0.2232010379623150 | 0.0479839333057554 |
|  | 34 | 0.0504792790607720 | 0.2232010379623150 | 0.2232010379623150 | 0.5031186450145980 | 0.0479839333057554 |
|  | 35 | 0.2500000000000000 | 0.2500000000000000 | 0.2500000000000000 | 0.2500000000000000 | 0.0931745731195340 |
|  | | | | | | |
| 6 | 1 | 0.9551438045408220 | 0.0149520651530592 | 0.0149520651530592 | 0.0149520651530592 | 0.0010373112336140 |
|  | 2 | 0.0149520651530592 | 0.9551438045408220 | 0.0149520651530592 | 0.0149520651530592 | 0.0010373112336140 |
|  | 3 | 0.0149520651530592 | 0.0149520651530592 | 0.9551438045408220 | 0.0149520651530592 | 0.0010373112336140 |
|  | 4 | 0.0149520651530592 | 0.0149520651530592 | 0.0149520651530592 | 0.9551438045408220 | 0.0010373112336140 |
|  | 5 | 0.7799760084415400 | 0.1518319491659370 | 0.0340960211962615 | 0.0340960211962615 | 0.0096016645399480 |
|  | 6 | 0.1518319491659370 | 0.7799760084415400 | 0.0340960211962615 | 0.0340960211962615 | 0.0096016645399480 |
|  | 7 | 0.7799760084415400 | 0.0340960211962615 | 0.1518319491659370 | 0.0340960211962615 | 0.0096016645399480 |
|  | 8 | 0.1518319491659370 | 0.0340960211962615 | 0.7799760084415400 | 0.0340960211962615 | 0.0096016645399480 |
|  | 9 | 0.7799760084415400 | 0.0340960211962615 | 0.0340960211962615 | 0.1518319491659370 | 0.0096016645399480 |
|  | 10 | 0.1518319491659370 | 0.0340960211962615 | 0.0340960211962615 | 0.7799760084415400 | 0.0096016645399480 |
|  | 11 | 0.0340960211962615 | 0.7799760084415400 | 0.1518319491659370 | 0.0340960211962615 | 0.0096016645399480 |
|  | 12 | 0.0340960211962615 | 0.1518319491659370 | 0.7799760084415400 | 0.0340960211962615 | 0.0096016645399480 |
|  | 13 | 0.0340960211962615 | 0.7799760084415400 | 0.0340960211962615 | 0.1518319491659370 | 0.0096016645399480 |
|  | 14 | 0.0340960211962615 | 0.1518319491659370 | 0.0340960211962615 | 0.7799760084415400 | 0.0096016645399480 |
|  | 15 | 0.0340960211962615 | 0.0340960211962615 | 0.7799760084415400 | 0.1518319491659370 | 0.0096016645399480 |
|  | 16 | 0.0340960211962615 | 0.0340960211962615 | 0.1518319491659370 | 0.7799760084415400 | 0.0096016645399480 |
|  | 17 | 0.3549340560639790 | 0.5526556431060170 | 0.0462051504150017 | 0.0462051504150017 | 0.0164493976798232 |
|  | 18 | 0.5526556431060170 | 0.3549340560639790 | 0.0462051504150017 | 0.0462051504150017 | 0.0164493976798232 |
|  | 19 | 0.3549340560639790 | 0.0462051504150017 | 0.5526556431060170 | 0.0462051504150017 | 0.0164493976798232 |
|  | 20 | 0.5526556431060170 | 0.0462051504150017 | 0.3549340560639790 | 0.0462051504150017 | 0.0164493976798232 |
|  | 21 | 0.3549340560639790 | 0.0462051504150017 | 0.0462051504150017 | 0.5526556431060170 | 0.0164493976798232 |
|  | 22 | 0.5526556431060170 | 0.0462051504150017 | 0.0462051504150017 | 0.3549340560639790 | 0.0164493976798232 |
|  | 23 | 0.0462051504150017 | 0.3549340560639790 | 0.5526556431060170 | 0.0462051504150017 | 0.0164493976798232 |
|  | 24 | 0.0462051504150017 | 0.5526556431060170 | 0.3549340560639790 | 0.0462051504150017 | 0.0164493976798232 |
|  | 25 | 0.0462051504150017 | 0.3549340560639790 | 0.0462051504150017 | 0.5526556431060170 | 0.0164493976798232 |
|  | 26 | 0.0462051504150017 | 0.5526556431060170 | 0.0462051504150017 | 0.3549340560639790 | 0.0164493976798232 |
|  | 27 | 0.0462051504150017 | 0.0462051504150017 | 0.3549340560639790 | 0.5526556431060170 | 0.0164493976798232 |
|  | 28 | 0.0462051504150017 | 0.0462051504150017 | 0.5526556431060170 | 0.3549340560639790 | 0.0164493976798232 |
|  | 29 | 0.5381043228880020 | 0.2281904610687610 | 0.2281904610687610 | 0.0055147549744775 | 0.0153747766513310 |
|  | 30 | 0.2281904610687610 | 0.5381043228880020 | 0.2281904610687610 | 0.0055147549744775 | 0.0153747766513310 |
|  | 31 | 0.2281904610687610 | 0.2281904610687610 | 0.5381043228880020 | 0.0055147549744775 | 0.0153747766513310 |
|  | 32 | 0.5381043228880020 | 0.2281904610687610 | 0.0055147549744775 | 0.2281904610687610 | 0.0153747766513310 |
|  | 33 | 0.2281904610687610 | 0.5381043228880020 | 0.0055147549744775 | 0.2281904610687610 | 0.0153747766513310 |
|  | 34 | 0.2281904610687610 | 0.2281904610687610 | 0.0055147549744775 | 0.5381043228880020 | 0.0153747766513310 |
|  | 35 | 0.5381043228880020 | 0.0055147549744775 | 0.2281904610687610 | 0.2281904610687610 | 0.0153747766513310 |
|  | 36 | 0.2281904610687610 | 0.0055147549744775 | 0.5381043228880020 | 0.2281904610687610 | 0.0153747766513310 |
|  | 37 | 0.2281904610687610 | 0.0055147549744775 | 0.2281904610687610 | 0.5381043228880020 | 0.0153747766513310 |
|  | 38 | 0.0055147549744775 | 0.5381043228880020 | 0.2281904610687610 | 0.2281904610687610 | 0.0153747766513310 |
|  | 39 | 0.0055147549744775 | 0.2281904610687610 | 0.5381043228880020 | 0.2281904610687610 | 0.0153747766513310 |
|  | 40 | 0.0055147549744775 | 0.2281904610687610 | 0.2281904610687610 | 0.5381043228880020 | 0.0153747766513310 |
|  | 41 | 0.1961837595745600 | 0.3523052600879940 | 0.3523052600879940 | 0.0992057202494530 | 0.0293520118375230 |
|  | 42 | 0.3523052600879940 | 0.1961837595745600 | 0.3523052600879940 | 0.0992057202494530 | 0.0293520118375230 |
|  | 43 | 0.3523052600879940 | 0.3523052600879940 | 0.1961837595745600 | 0.0992057202494530 | 0.0293520118375230 |
|  | 44 | 0.1961837595745600 | 0.3523052600879940 | 0.0992057202494530 | 0.3523052600879940 | 0.0293520118375230 |
|  | 45 | 0.3523052600879940 | 0.1961837595745600 | 0.0992057202494530 | 0.3523052600879940 | 0.0293520118375230 |
|  | 46 | 0.3523052600879940 | 0.3523052600879940 | 0.0992057202494530 | 0.1961837595745600 | 0.0293520118375230 |
|  | 47 | 0.1961837595745600 | 0.0992057202494530 | 0.3523052600879940 | 0.3523052600879940 | 0.0293520118375230 |
|  | 48 | 0.3523052600879940 | 0.0992057202494530 | 0.1961837595745600 | 0.3523052600879940 | 0.0293520118375230 |
|  | 49 | 0.3523052600879940 | 0.0992057202494530 | 0.3523052600879940 | 0.1961837595745600 | 0.0293520118375230 |
|  | 50 | 0.0992057202494530 | 0.1961837595745600 | 0.3523052600879940 | 0.3523052600879940 | 0.0293520118375230 |
|  | 51 | 0.0992057202494530 | 0.3523052600879940 | 0.1961837595745600 | 0.3523052600879940 | 0.0293520118375230 |
|  | 52 | 0.0992057202494530 | 0.3523052600879940 | 0.3523052600879940 | 0.1961837595745600 | 0.0293520118375230 |
|  | 53 | 0.5965649956210170 | 0.3523052600879940 | 0.3523052600879940 | 0.1344783347929940 | 0.0366291366405108 |
|  | 54 | 0.1344783347929940 | 0.5965649956210170 | 0.3523052600879940 | 0.1344783347929940 | 0.0366291366405108 |
|  | 55 | 0.1344783347929940 | 0.1344783347929940 | 0.5965649956210170 | 0.1344783347929940 | 0.0366291366405108 |
|  | 56 | 0.1344783347929940 | 0.1344783347929940 | 0.1344783347929940 | 0.5965649956210170 | 0.0366291366405108 |
